# A Focus on Abuse/Misuse and Withdrawal Issues with Selective Serotonin Reuptake Inhibitors (SSRIs): Analysis of Both the European EMA and the US FAERS Pharmacovigilance Databases

**DOI:** 10.3390/ph15050565

**Published:** 2022-05-01

**Authors:** Stefania Chiappini, Rachel Vickers-Smith, Amira Guirguis, John Martin Corkery, Giovanni Martinotti, Fabrizio Schifano

**Affiliations:** 1 Psychopharmacology, Drug Misuse and Novel Psychoactive Substances Research Unit, School of Life and Medical Sciences, University of Hertfordshire, London AL10 9EU, UK; stefaniachiappini9@gmail.com (S.C.); j.corkery@herts.ac.uk (J.M.C.); giovanni.martinotti@gmail.com (G.M.); f.schifano@herts.ac.uk (F.S.); 2 Department of Epidemiology, College of Public Health, University of Kentucky, 111 Washington Avenue, Lexington, KY 40536, USA; 3 Swansea University Medical School, The Grove, Swansea University, Swansea SA2 8PP, UK; amira.guirguis@swansea.ac.uk; 4 Department of Neurosciences, Imaging and Clinical Sciences, Università degli Studi “G. d’Annunzio” Chieti-Pescara, 66100 Chieti, Italy

**Keywords:** antidepressants, selective serotonin reuptake inhibitor (SSRI), withdrawal, prescription drug abuse, drug misuse, pharmacovigilance

## Abstract

Despite increasing reports, antidepressant (AD) misuse and dependence remain underestimated issues, possibly due to limited epidemiological and pharmacovigilance evidence. Thus, here we aimed to determine available pharmacovigilance misuse/abuse/dependence/withdrawal signals relating to the Selective Serotonin Reuptake Inhibitors (SSRI) citalopram, escitalopram, paroxetine, fluoxetine, and sertraline. Both EudraVigilance (EV) and Food and Drug Administration-FDA Adverse Events Reporting System (FAERS) datasets were analysed to identify AD misuse/abuse/dependence/withdrawal issues. A descriptive analysis was performed; moreover, pharmacovigilance measures, including the reporting odds ratio (ROR), the proportional reporting ratio (PRR), the information component (IC), and the empirical Bayesian geometric mean (EBGM) were calculated. Both datasets showed increasing trends of yearly reporting and similar signals regarding abuse and dependence. From the EV, a total of 5335 individual ADR reports were analysed, of which 30% corresponded to paroxetine (*n* = 1592), 27% citalopram (*n* = 1419), 22% sertraline (*n* = 1149), 14% fluoxetine (*n* = 771), and 8% escitalopram (*n* = 404). From FAERS, a total of 144,395 individual ADR reports were analysed, of which 27% were related to paroxetine, 27% sertraline, 18% citalopram, 16% fluoxetine, and 13% escitalopram. Comparing SSRIs, the EV misuse/abuse-related ADRs were mostly recorded for citalopram, fluoxetine, and sertraline; conversely, dependence was mostly associated with paroxetine, and withdrawal to escitalopram. Similarly, in the FAERS dataset, dependence/withdrawal-related signals were more frequently reported for paroxetine. Although SSRIs are considered non-addictive pharmacological agents, a range of proper withdrawal symptoms can occur well after discontinuation, especially with paroxetine. Prescribers should be aware of the potential for dependence and withdrawal associated with SSRIs.

## 1. Introduction

Due to their demonstrated efficacy, antidepressants (AD) play a key role in the treatment of both mood and anxiety disorders [1]. The last 20 years of data from the Medical Expenditure Panel Survey, the nationally representative database of the United States/US population, described a substantial increase in long-term AD prescriptions, with the selective serotonin reuptake inhibitors (SSRIs) sertraline, fluoxetine, citalopram, escitalopram, and paroxetine having been the most popular AD among prescribers [2,3,4]. Similar trends relating to the use of SSRIs have been reported by both European countries [5,6,7,8] and the United Kingdom (UK), where, in 2017–2018, 7.3 million people (i.e., 17% of the adult population) were prescribed with an AD [9].

SSRIs are generally well tolerated and considered to be safer than earlier ADs such as most tricyclic antidepressants (TCAs) and monoamine oxidase inhibitors (MAOIs) [1,10]. However, recent evidence shows that SSRIs are associated with a withdrawal reaction upon the abrupt discontinuation of long-term use of regular/high doses [11,12]. A further emerging problem is that SSRIs may themselves be entering the repertoire of polydrug users [1,13,14]. Drug abuse has been increasingly reported in the past ten years, relating to both several prescriptions (e.g., quetiapine, pregabalin, gabapentin, etc.) and over-the-counter (OTC) drugs (e.g., loperamide, dextromethorphan, promethazine, etc.), traditionally considered devoid of abuse liability [13,14,15,16,17].

### 1.1. Abusing with an AD

Both MAOIs, and especially those with amphetamine-like structures, and the TCA amitriptyline have been associated with misuse/abuse/dependence/withdrawal-related issues [18,19,20]. Furthermore, recreational ingestion of bupropion (e.g., through nasal insufflation or intravenous injection of crushed tablets) has been associated with a cocaine-like “high” [21,22,23], and the intake of high-dosage venlafaxine (“baby ecstasy”) to achieve an “amphetamine-like high” [1,16,21,24] has been described. AD abuse has been shown to typically occur among both inmates, where specific substances have been removed from some correctional facilities’ formularies [1], and among clients with comorbid substance use and mood disorders [1,10]. Whilst SSRIs are generally considered not to possess any abuse liability, a few case reports/series of their misuse [1] have identified the intake of fluoxetine and sertraline by those taking 3,4 methylenedioxymethamphetamine (MDMA) at clubs to prolong the “high” from 2 to 4 h and make the “come down” easier [25].

### 1.2. AD and SSRI-Associated Withdrawal Issues

The rate of people experiencing some degree of withdrawal effects upon cessation of AD is within 55–65%; the molecules mentioned most often involve paroxetine, escitalopram, venlafaxine, and TCAs, with the withdrawal clinical syndrome being severe in nearly half (46%) of cases [26,27]. In particular, and despite their popularity, there is a relative lack of awareness about the likely underestimated phenomenon [26,27,28,29,30,31] of the SSRIs’ withdrawal effects. Related signs and symptoms range from increased anxiety and hyperarousal, sensory disturbances, and psychological manifestations such as agitation, dysphoria, hallucinations, and confusion [10,26,29,32,33,34]. Current US and UK clinical guidelines indicate that withdrawal reactions are usually self-limiting over about 1–2 weeks [31,35]; however, symptoms may appear up to 10 days after having stopped/reduced the index SSRI dosage [29] and can persist for a longer period [26,34,35,36]. The withdrawal may be more likely to be observed with short half-life/high potency SSRIs, such as paroxetine, and unlikely with the long half-life fluoxetine [10,34,37,38].

### 1.3. AD and SSRI-Associated Withdrawal Issues; Post-Marketing Evidence

The French drug surveillance database, supported by the French National Agency of Medicine, was queried in 1997 for neuro-psycho behavioural reactions associated with SSRIs; similar safety profiles were identified for fluoxetine, fluvoxamine, and paroxetine. Conversely, withdrawal reactions, respectively, at 13% and 14%, were more common with fluvoxamine and paroxetine compared with the 1.5% relating to fluoxetine [39]. Similarly, data from the UK Yellow Card Scheme (YCS) recorded a greater proportion of withdrawal reactions with paroxetine (5.1%) compared with other SSRIs (0.06–0.9%) [40]. An analysis of 1374 emails following the “Secrets of Seroxat” BBC-TV programme and of 862 emails collected from the website ADWEB found that the high number of paroxetine adverse drug reactions (ADRs) were possibly attributable to both the drug’s dominant market position and to its relatively short half-life [41]. Finally, paroxetine and venlafaxine, in comparison with fluoxetine and bupropion, were found to be more frequently associated with AD abuse- and dependence-related ADRs in both the EMA EudraVigilance (EV) and the YCS [21].

Aim of the study: The present study aimed at analysing two pharmacovigilance datasets, i.e., the EV and the FDA Adverse Event Reporting System (FAERS), in order to determine available pharmacovigilance misuse/abuse/dependence/withdrawal signals relating to the SSRIs citalopram, escitalopram, paroxetine, fluoxetine, and sertraline.

## 2. Results

### 2.1. EMA Dataset

During February 2003–April 2018, a total of 6102 ADR reports involving the selected ADs were submitted to the EV. We removed duplicates, observations missing the EV Local Report Number, cases where one of the selected AD drugs was not listed as a “suspect” cause of the index ADR case, and ADRs that listed multiple of the selected ADs. A total of 5335 individual ADR reports were included in the present analysis, of which 30% corresponded to paroxetine (*n* = 1592), 27% citalopram (*n* = 1419), 22% sertraline (*n* = 1149), 14% fluoxetine (*n* = 771), and 8% escitalopram (*n* = 404) (Table 1). 

**Figure 1:**
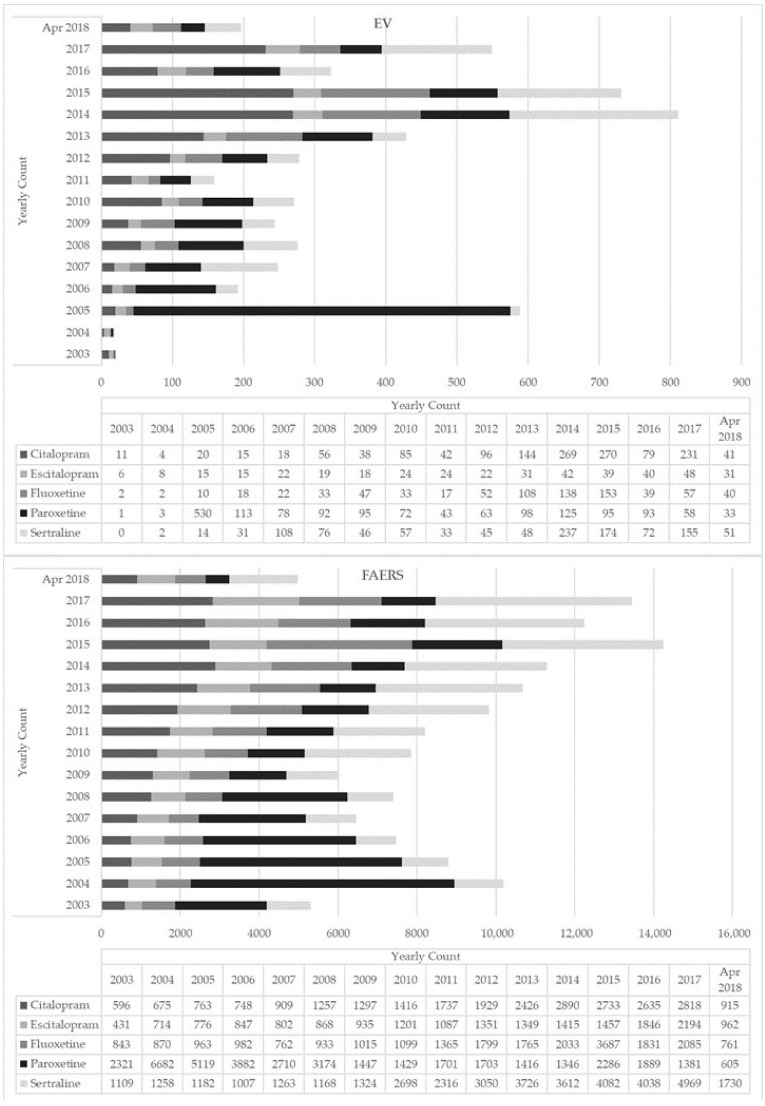
Yearly count of selective serotonin reuptake inhibitor (SSRI)-related adverse drug reactions reported to EudraVigilance (EV) and the Food and Drug Administration (FDA) Adverse Event Reporting System (FAERS) (2003–April 2018).

There was an increasing trend in ADR reporting every year for all five ADs with peaks in 2014 (Figure 1). The majority of the ADR reports for all ADs involved adult females (mean age 41.4–43.3 years) (Table 1); most reports came from the US and European countries (17%), except for paroxetine reports which, interestingly, primarily came from Japan (Table S1). Where reported, the majority of indications for all selected ADs were depression, anxiety, and drug abuse (Table 1). For all ADs, most instances (ranging from 62% to 82% of cases, depending on the index molecule) reported an oral route of administration (ROA). Interestingly, although not often (i.e., <1% of cases), a nasal ROA was reported for all ADs except escitalopram (Table 1). Concomitant drugs most commonly listed in the ADR reports included opioids and benzodiazepines, particularly with citalopram and fluoxetine. Additional concomitant drugs included: other ADs, antihistamines, antipsychotics, gabapentinoids, mood stabilizers, and Z-drugs (e.g., zaleplon, zolpidem, zopiclone). Recreational drugs most typically reported in combination with the selected ADs were cocaine and alcohol (Table 1). Fatal outcomes were most commonly recorded for citalopram (70% of cases), fluoxetine (55%), and sertraline (46%) (Table 2).

With respect to the other SSRIs, misuse/abuse- related ADRs were most often recorded for citalopram, fluoxetine, and sertraline (Table S2). Specifically, significant pharmacovigilance signals for “drug abuse” were identified for citalopram, fluoxetine, and sertraline. Compared to the other selected ADs, “drug abuse” was listed as an ADR more than four times as frequently for citalopram (proportional reporting ratio [PRR] = 4.12) and nearly twice as frequently for both fluoxetine (PRR = 1.77) and sertraline (PRR = 1.57; all false discovery rates [FDR] < 0.01). With regard to dependence-related ADRs, significant signals were identified primarily for paroxetine (Table S2); “dependence” was reported for paroxetine more than six times as frequently (PRR = 6.45) and “drug dependence” reported nearly twice as often (PRR = 1.84; all FDR < 0.01) for paroxetine compared to the other ADs. For withdrawal ADR reports, “drug withdrawal syndrome” was recorded nearly twice as often for escitalopram compared to the other ADs (PRR = 1.68; FDR < 0.01).

Other significant drug ADR signals identified were (all FDR < 0.01): citalopram and “delirium” (PRR = 2.09), escitalopram and “somnolence” (PRR = 1.81), paroxetine and “aggression” (PRR = 3.02), and sertraline and “feeling abnormal” (PRR = 1.71) (Table S3).

### 2.2. FAERS Dataset

During February 2003–April 2018, a total of 302,330 ADR reports involving the selected ADs were submitted to the FAERS database. After removing duplicates and other observations as described in Section 2.2 for the EV database, a total of 144,395 individual ADR reports were included in the present analysis, of which 27% were related to paroxetine, 27% sertraline, 18% citalopram, 16% fluoxetine, and 13% escitalopram (Table 1). While number of reports increased overtime for all ADs, fluoxetine showed a peak in 2015 (Figure 1).

Where reported, the majority of indications for all selected ADs were depression, anxiety, and drug abuse (Table 1). For all selected ADs, most reports came from the US and a range of European countries, e.g., UK, France, and Italy, although for paroxetine and sertraline reports, Japan featured among the top five countries from which reports were received (Table S1). For all ADs, most instances (ranging from 57% to 78% of cases, depending on the index molecule) reported an oral ROA. Although not often, unusual ROAs were listed such as intravenous (Table 1). Concomitant drugs most commonly listed in the ADR reports were benzodiazepines, opioids, antipsychotics, and other ADs; alcohol was the most commonly reported recreational substance (Table 1). Fatal outcomes were often recorded: citalopram (29%), fluoxetine (20%), escitalopram (13%), sertraline (13%), and paroxetine (9%) (Table 2).

Significant pharmacovigilance signals for misuse/abuse-related ADRs were identified primarily for citalopram and fluoxetine (Table S2). Specifically, the ADR “drug abuse” was listed more than three times as frequently for citalopram (PRR = 3.35) and 1.2 times as often for fluoxetine (PRR = 1.22) compared to the other ADs (all FDR < 0.01). The ADR “drug diversion” was reported more than three times as often for sertraline compared to the other ADs (PRR = 3.11; FDR < 0.01). The ADRs “drug withdrawal syndrome”, “drug dependence”, and “dependence” were reported much more often for paroxetine than the other ADs (PRRs = 13.68, 3.61, and 27.42, respectively; all FDR < 0.01) (Table S2).

Other significant drug ADR signals identified were (all FDR < 0.01): citalopram and “ataxia” (PRR = 2.31), escitalopram and “fall” (PRR = 1.55); fluoxetine and “mixed hallucinations” (PRR = 1.82), paroxetine and “dissociation” (PRR = 2.63; FDR < 0.01), and sertraline and “substance-induced psychotic disorder” (PRR = 6.93; FDR < 0.01) (Table S3).

## 3. Discussion

To the best of our knowledge, this is the most comprehensive pharmacovigilance analysis of SSRI misuse/abuse/dependence and withdrawal issues. A total number of 149,730 unique individual cases/patients, including 5335 from the EV dataset and 144,395 from the FAERS, were here identified.

### 3.1. Comparison between the Two Datasets

Both datasets were consistent in terms of the most recorded ADs which included, in descending order, paroxetine, sertraline, and citalopram. EV and FAERS data were also comparable in terms of the most reported gender and age characteristics of patients involved, reflecting current information on AD use [8]. Both dataset entries were typically originating from the US and European countries, although large numbers of ADRs related to sertraline and paroxetine were recorded from Japan. One could argue that this large volume of entries from a single country is probably related to a recently growing awareness of pharmacovigilance and drug safety risk assessment, in parallel with the launch of the Japanese Adverse Drug Event Report (JADER) system’s free access/free use in 2012 [42,43,44]. Concomitant drugs prescribed with ADs in both the EV and FAERS datasets included benzodiazepines and opioids, e.g., molecules that are typically prescribed on a chronic basis [45,46,47]. Data regarding ADs’ dispensed prescriptions suggest a long-term (>12 months) prescribing pattern as well [2,28], despite this approach being debatable [27,48]. Antihistamines, often considered for the treatment/management of sleeping disorders [49], were frequently reported in AD ADRs. However, reports of these molecules’ abuse and diversion, either on their own or in association with ADs/other drugs, have been made available [15,17,50,51]. In both the EV and the FAERS datasets, antihistamines were found to be most typically associated with citalopram and fluoxetine, possibly because these molecules possess only limited sedating properties with respect to remaining SSRIs [52]. Cocaine and alcohol were frequently reported here in combination with the SSRIs. Substance misuse and depression are both highly prevalent, frequently co-occurring, conditions [53,54]. SSRI medications, and especially sertraline, are being used alone, or in combination, for the treatment of people with co-occurring depression and drug/alcohol dependence, although the clinical relevance of this approach may be limited [55].

### 3.2. SSRIs Abusing Issues; Differences between the Molecules Examined

From both datasets, the abuse-related signals were here mostly recorded in association with citalopram and fluoxetine, and to a lesser extent with sertraline. This finding is consistent with data from the US Researched Abuse, Diversion, and Addiction-Related Surveillance (RADARS) System, suggesting that the most common non-scheduled psychoactive prescription drugs diverted over a 16-year period included sertraline, fluoxetine, and citalopram, along with other psychotropics [56]. Despite being generally considered a safe class [57], there is a growing, albeit relatively small, literature reporting the misuse and abuse of SSRIs; many of these reports involved fluoxetine, ingested in idiosyncratic ways (e.g., intravenously) and/or at mega-dosages (e.g., up to 120 mg), for either appetite suppression/weight loss or for stimulant-like effects in patients with a substance use history [1]. Conversely, whilst citalopram and sertraline are less frequently reported in association with misusing/abusing issues, they have both been identified in overdose-related arrhythmias [58,59,60,61]. To this respect, it is worth noting that euphoric mood, which may in itself be associated with a recreational drug-related “high” [62], was one of the most recorded PTs associated with both fluoxetine and sertraline.

There are similarities related to all molecules pertaining to the SSRI class; all of them boost the neurotransmitter serotonin/5HT through a blockade of the serotonin reuptake pump. This is associated with both a desensitisation of the serotonin receptors, especially serotonin 1A, and overall increasing levels of the serotonergic neurotransmission. However, citalopram, fluoxetine, and sertraline show several differences in terms of potency and selectivity. Indeed, citalopram seems to represent the most selective inhibitor of 5HT uptake, having minimal effects on dopamine and noradrenaline transporters and mild antagonist actions at H1 histamine receptors; fluoxetine shows antagonist properties at 5HT2C receptors, which could increase noradrenaline and dopamine neurotransmission; and, finally, sertraline may possess some ability to block the dopamine transporter, hence increasing dopamine neurotransmission, whilst also binding at sigma 1 receptors [52]. Despite an abuse liability of these three SSRIs having not been previously suggested, and the related pharmacological mechanisms might not yet be clear, several and complex factors might influence the possible diversion and abuse/misuse of SSRIs. It is generally accepted that drugs with addictive properties act on brain systems subserving reinforcement or reward and involving both multiple brain areas and multiple neurotransmitters. The most important one is the dopaminergic mesocorticolimbic pathway, probably underlying the positive motivational or incentive aspects of reward- and of drug-seeking behaviour (for an overview, see [62]). Further interacting systems postulated to be involved in rewarding actions are those related to endogenous opioids; the GABAergic system, involved with substances such as alcohol, barbiturates, and benzodiazepines; and a few others, such as the noradrenaline, cholecystokinin, glutamate, and neuropeptide Y pathways [63]. Serotonin appears to play a dual role in reward; in fact, both the ventral tegmental area and the nucleus accumbens receive serotonergic projections from the dorsal and median raphe nuclei. The serotonergic activity in the ventral tegmental area appears to be excitatory, resulting in increased levels of dopamine release in the nucleus accumbens [63]. A second point to be considered is the possibility of a current/previous history of substance abuse in patients reported here to have misused SSRIs. In fact, the non-medical use of SSRIs might occur in people using medicines without medical reasons either for recreational purposes or for reducing withdrawal/adverse symptoms occurring after having ingested other recreational psychotropics [64]. Unfortunately, current data may only be of partial help; in fact, in the citalopram, escitalopram, and fluoxetine EV cases, “drug abuse” was mentioned as a clinical indication, consistent with previous literature suggestions [55,65]. Despite this, people who use drugs may represent a vulnerable population when being prescribed with any AD [1]. At present, no evidence-based guidelines for the treatment and management of individuals with comorbid mood and substance use disorders, and specifically of depressed subjects misusing ADs, are available. A careful history and risk stratification assessment, including a history of legal, prescribed, and illicit drug abuse, is an important strategy for reducing the likelihood of AD misuse when evaluating a new patient. Finally, although SSRIs are thought to be relatively safe in overdose [66,67] a range of fatal reports were recorded here with citalopram, fluoxetine and less frequently with sertraline. Apart from those cases where an intentional overdose with suicide intent occurred [68,69], SSRI-related fatalities are relatively rare. In this respect, some risk factors have been identified, including the concurrent ingestion of (i) sedatives such as alcohol, benzodiazepines, and opioids; (ii) drugs that can facilitate the occurrence of a serotonin toxicity, e.g., tramadol and amphetamines; and (iii) other drugs involved in CYP-mediated drug–drug interactions, since fluoxetine and paroxetine are potent CYP2D6 inhibitors [1,60,70].

### 3.3. SSRIs’ Dependence and Withdrawal Issues; Clinical and Theoretical Considerations

Current findings, suggesting high levels of paroxetine-related dependence/withdrawal issues in comparison with remaining SSRIs, are consistent with previous literature suggestions [8,27,30,39,71]. Conversely, due to its long half-life, fluoxetine is not typically associated with withdrawal signs/symptoms even when abruptly discontinued; furthermore, sertraline, citalopram, and escitalopram all present with a low risk of withdrawal symptoms [12,18,26,27,40,72,73,74]. Paroxetine metabolism is linked to cytochrome CYP2D6 [63,64]. At high concentrations, paroxetine inhibits CYP2D6, slowing its own inactivation; hence, a dose increase might lead to a disproportionate increase in plasma levels. Conversely, abruptly stopping the drug could cause a sharp drop in plasma levels, which may help explain the withdrawal symptoms’ intensity [74,75,76,77].

When discussing both SSRI-related dependence and withdrawal, which is a more appropriate term than “discontinuation” [12,78], some issues may, however, need to be considered. Dependence is characterised per se by tolerance and/or withdrawal symptoms, with “withdrawal”, however, not necessarily including the occurrence of physical signs and symptoms. Finally, “addiction” is characterised by a further range of issues, e.g., compulsive substance use; craving; and continued use despite its adverse consequences (for an overview, see [36]). Hence, withdrawal symptoms that occur upon the discontinuation of medications prescribed do not suggest, per se, either a substance-related [79] or an addiction disorder [80]. This may well be the case with ADs, including the SSRIs [12]. Syndromes of withdrawal occurring with most recreational and a range of prescribed drugs may include the following features: (a) rebound, e.g., the re-occurrence of the original symptoms for which the index medication was prescribed; (b) withdrawal properly called, including both rebound and new unrelated symptoms; and (c) persistent post-withdrawal disorder, characterised by a return of the original illness at higher severity (for an overview of the issue, see [81]). The recently proposed “oppositional model” of tolerance [71], the concept of behavioural toxicity [36,70], and the SSRI-related counter adaptive neuro-regulation effects [82] can help in explaining the potential onset of an AD discontinuation-related withdrawal/persistent post-withdrawal disorder. Other related issues of clinical relevance include relapse, considered the re-emergence of the same disease episode due to loss of pharmacological effects, and recurrence intended as a new episode of a recurring primary disorder following previous recovery (e.g., a remission over 6–9 months) due to the loss of pharmacological effect [12,26,74].

Hence, although SSRIs are considered non-addictive pharmacological agents, a range of proper withdrawal symptoms can occur well after discontinuation. Indeed, when tapering down a therapeutic dosage of AD, symptoms most typically are both mild/go untreated and resolve spontaneously [81,83]. A number of these symptoms may resemble the primary disease (e.g., depression, anxiety, irritability), whereas others can be clearly differentiated from the disorder, with most common symptoms including flu-like symptoms; disturbed sleep and vivid dreams/nightmares; imbalance/dizziness/light-headedness; nausea; and sensory disturbances, e.g., electric shock-like sensations and dysesthesia [40,74]. Indeed, most of these signs and symptoms were here described as paroxetine withdrawal-related PTs. Others [74] have also suggested that a range of withdrawal symptoms may indeed relate to the occurrence of a serotonin syndrome; SSRIs can in fact facilitate not only the blockade of serotonin transporters, but also their reduction/down-regulation after long-term use, resulting in serotonin hyperfunction after the SSRI isdiscontinued. Finally, in cases where SSRIs were ingested at mega, as opposed to therapeutic, dosage levels, similar to what occurs when either gabapentinoids or benzodiazepines are discontinued, the associated withdrawal, persistent post-withdrawal, and overall behavioural toxicity issues may be particularly relevant [36] and need proper long-term specialist attention [36]. Hence, if an AD has been used for several months/years, its slow dosage tapering down should be considered. If the patient complains of clear clinical signs/symptoms of withdrawal, maintaining the previous AD dosage or adding a new treatment such as a mood stabilizer or a benzodiazepine to support the AD reduction may be considered useful strategies [29].

### 3.4. The “Denominator” Issue; Focus on SSRIs’ Prescription Data

The increasing rates in relating reporting overtime here identified may suggest a recently growing emphasis on pharmacovigilance data [18,84,85], which may well provide both real-world and affordable information on medications’ use/misuse beyond what is normally recorded in controlled trials [15]. Consistent with this, prescription-based methods of drug safety surveillance might represent areas of possible progress by combining aspects of public health surveillance, spontaneous reporting, and epidemiological studies [86]. The great advantage of this approach is that it would provide a numerator (e.g., the number of reports) and a denominator (e.g., the number of patients exposed), both being collected over a precisely known period of observation [86,87]. One could argue that the increasing number of reports over time observed herein was associated with a rise in AD prescribing. Unfortunately, however, detailed prescription data are typically available only at a national level [2,28,88,89], whereas both the EV and the FAERS collect data at an international, cross-countries level [90,91]. Worldwide, overall prescription data may indeed suggest increasing levels of both depression diagnoses being made and AD prescriptions; the most popular molecules would be sertraline, followed by fluoxetine, citalopram, and escitalopram [2,7,28].

With the lack of reliable worldwide prescription data, a representative sample of national data from the Prescription Cost Analysis (PCA), providing freely available numbers of all prescriptions dispensed in the community in England, was here considered [92]. PCA data showed that citalopram was the most prescribed AD, whilst sertraline prescriptions have risen rapidly, overtaking paroxetine (Figure S1). The total number of PCA annual prescription items showed a continuous rise during years 2004–2018, and especially so for the single ADs citalopram, fluoxetine, and sertraline, while paroxetine gradually reduced over years, and escitalopram remained almost stable (Figure S1). These observations are consistent with findings from a retrospective analysis of anonymised data on medicines prescribed by general practitioners (GPs) in England from the Open-Prescribing Database [93] and with current findings, showing that paroxetine ADRs reduced over the years, whilst citalopram, fluoxetine, and sertraline showed a peak in 2014–2015 (Figure 1). From the US, results from the last National Health and Nutrition Examination Survey (NHANES) from the National Center for Health Statistics, providing the estimate number of individuals receiving a certain type of medication in the past month, were here analysed to evaluate trends in SSRI use [94,95,96]. Although the size of data relating to each of the five SSRIs here examined was too small to be analysed, a consistent overall rise in the US prevalence of AD use over the years 2003–2018, with a peak during years 2011–2012 and 2013–2014, was observed (Table S4).

### 3.5. Limitations

Whilst disproportionality analysis may be a suitable tool to quantify signals of drug abuse, it presents, however, with a limited capacity to differentiate the type (e.g., recreational; self-medication; etc.) [97]. In addition, confounding factors such as comorbidity and concomitant drugs cannot be assessed properly with a pharmacovigilance approach. Moreover, although care was taken to remove duplicates based on the report identification number, duplicate records may still exist in the data (i.e., different identification numbers, but similar data) due to an overlap difference between datasets, e.g., the relative number of EU cases in EV and the ratio of EV cases and FAERS cases, presumably due to differences in marketing authorizations or market penetration in different regions [98]. Finally, the study of ADRs alone is rarely sufficient to confirm that a certain effect in a patient has been caused by a specific medicine. In fact, a drug-related adverse reaction reported does not necessarily mean that the specific medicine has caused the observed effect, as this could have also been caused by the disease being treated, a new disease the patient developed, or by another medicine that the patient is taking. Single case reports reflect the information as provided to EV or to the FDA by the reporter. Thus, a single case report should only be regarded as a piece of information, with further data (e.g., worldwide spontaneous case reports, clinical trials, and epidemiological studies) being needed to obtain a full understanding of the safety profile of an index molecule. Thus, both the EMA and the FAERS data by themselves are not an indicator of the safety profile of a drug. Indeed, the number of case reports for a particular medicine or suspected adverse reaction does not only depend on the real frequency of the adverse reaction but also on a number of external factors influencing spontaneous reporting such as the extent and condition of use of the medicine, the nature of the reaction, public awareness [91], and others, e.g., the “ripple effect”, where reporting is accelerated following the publicity of a drug in the same class, or the “notoriety effect”, where there is an increase in reporting resulting from a safety alert [99]. Another limitation may be related to the choice of the molecules being here investigated, a choice which did not include all SSRIs. Indeed, citalopram, escitalopram, fluoxetine, paroxetine, and sertraline were selected whilst considering their first clinical indication, which is major depression. Consistent with this, both fluvoxamine and dapoxetine were here excluded. In fact, whilst fluvoxamine is commonly prescribed in the UK and in most European countries to treat major depression and obsessive-compulsive disorder, in the US it is approved by the FDA for the treatment of obsessive-compulsive and social anxiety disorders only. Conversely, dapoxetine is an SSRI prescribed in some countries for the treatment of premature ejaculation only [6,18,52,88].

## 4. Materials and Methods

### 4.1. Data Sources

The EMA is responsible for the EudraVigilance (EV) recording of ADRs reported for all medicinal products authorised in the European Economic Area (EEA) [91]. For the present study, we requested data from the EMA in April 2018 for ADR reports for the selected ADs submitted to the EV during 2003–present. All reports included cases where fluoxetine, paroxetine, citalopram, escitalopram, or sertraline were reported as a suspected or interacting active substance. Preferred terms (PT) for the present analysis were selected from the standardised Medical Dictionary for Regulatory Activities (MedDRA) Query (SMQ) including “Drug abuse, dependence and withdrawal” [100] including “Drug abuse”, “Substance abuse”, “Intentional product misuse”, “Dependence”, “Drug withdrawal syndrome”, “Withdrawal”, and “Withdrawal syndrome”. PTs that may be indicative of an abuse event (described in detail in [101]) were also examined in this analysis.

Similarly, the FAERS, designed to support the FDA’s post-marketing safety that contains information on adverse event and medication error reports submitted to the FDA [90], was queried in April 2018 for ADRs related to the selected Ads. FAERS data were available through the FAERS Public Dashboard and quarterly data extract files [90]. To enable a clearer comparison between EV and FAERS, we used the same timeframe for both datasets in the present analysis; therefore, any ADRs occurring in FAERS prior to February 2003 (the date of the first EV ADR for one of the five SSRIs under investigation herein) were removed from the analysis. In the present study, misuse is defined as “the intentional and inappropriate use of a product other than as prescribed or not in accordance with the authorized product information” [36]; abuse is “the intentional, non-therapeutic, use by a patient or consumer of a product, over-the-counter/OTC or prescription, for a perceived reward or desired non-therapeutic effect including, but not limited to, getting high (euphoria)”; dependence is the “overwhelming desire by a patient or consumer to take a drug for non-therapeutic purposes together with inability to control or stop its use despite harmful consequences” [93]; and withdrawal is “the abrupt cessation or reduction in intake of a drug in a habituated person, resulting in a substance-specific syndrome, with symptoms varying according to the psychoactive substance used and generally opposite the acute effects of drug” [93].

### 4.2. Data Analysis

We performed a descriptive analysis of ADR report characteristics including sociodemographics, country of origin, most common diagnoses, ROA, and concomitant licit/illicit substances. SPSS^®^ v28 (Armonk, NY, USA: IBM Corp, 2017) was used for all descriptive analysis. Pharmacovigilance signal measures, including the reporting odds ratio (ROR), proportional reporting ratio (PRR), information component (IC), and empirical Bayesian geometric mean (EBGM), were calculated in each dataset using the R^®^ package PhViD [102]. All four pharmacovigilance measures were calculated due to differences in their sensitivity and early detection potential [18,101,103,104,105,106]; for brevity, only the PRR is shown in the text; all calculated measures can be found in the supplemental tables. Signals are disproportionality measures based on a 2 × 2 contingency table; they help determine whether a drug adverse event pair occurs more often than expected by comparing signal values to published thresholds [107,108]. Given the support for the use of the false discovery rate (FDR) to identify signals over thresholds, we used an FDR < 0.05 to denote significance [108]. When significant signals were reported in this analysis, all four measures met significance criteria.

Data from the PCA were extracted to determine the annual numbers of citalopram, escitalopram, fluoxetine, paroxetine, and sertraline prescriptions dispensed in the community in England from 2004 to 2018 [92]. To determine the estimated US prevalence of the selected SSRIs, data from the Demographic Variables and Sample Weights and Prescription Medications questionnaires of the 2003/2004 to 2017/2018 NHANES were downloaded [94]. Key drugs were identified by via their generic name in the RXDDRUG variable. To calculate the prevalence estimate and 95% confidence interval, we ran a Complex Samples analysis in SPSS^®^ v28 using the masked variance pseudo-stratum (SDMVSTRA) as the strata, masked variance pseudo-PSU (SDMVPSU) as the clusters, and full sample 2-year interview weight (WTINT2YR) as the sample weight. This was repeated for each annual questionnaire. We ensured that the sample size was large enough for the proportion and design effect based on tables provided from NHANES [109,110,111].

## 5. Conclusions

In this study, disproportionality signals of abuse/misuse/dependence and withdrawal related to several SSRIs have been shown. Although further and specific studies are needed to confirm these findings, in consideration of the common use of SSRIs, prescribers should be cautious in prescribing SSRIs and carefully evaluate the risk for some clients to be prone to ingest high/mega dosages of medications, often in combination with alcohol and illicit drugs. A rational and safe use of medicines incorporates the evaluation of all potential benefits and harms and their application only to cases indicated. Medication use should be limited to both the shortest possible time and the lowest dosage in order to avoid drug toxicity, in general, but also withdrawal and dependence issues; both augmentation strategies and psychotherapy may need to be considered in the long-term treatment of depressive and anxiety disturbances.

## Figures and Tables

**Table 1:** Analysis of suspect selective serotonin reuptake inhibitor (SSRI)-related adverse drug reactions reported to the European Medicines Agency (EMA) EudraVigilance (EV) dataset and the Food and Drug Administration (FDA) Adverse Event Reporting System.

	Citalopram		Escitalopram		Fluoxetine		Paroxetine		Sertraline	
	EMA	FAERS	EMA	FAERS	EMA	FAERS	EMA	FAERS	EMA	FAERS
**Individual Cases**	**1419**	**25,744**	**404**	**18,235**	**771**	**22,793**	**1592**	**39,091**	**1149**	**38,532**
**Mean Age in years (SD)**	42.6 (14.2)	47.6 (21.5)	43.3 (18.6)	48.2 (22.5)	43.1 (15.5)	42.5 (20.6)	41.4 (15.8)	44.0 (23.5)	41.6 (16.5)	45.6 (22.0)
**M/F (%)**	615/773 (44%/56%)	8770/14,169 (38%/62%)	138/244 (36%/64%)	5988/10,920 (35%/65%)	279/457 (38%/62%)	6547/13,141 (33%/67%)	554/959 (37%/63%)	13,124/22,609 (37%/63%)	493/606 (45%/55%)	12,245/21,972 (36%/64%)
**Most common psychiatric indications recorded for the index SSRI (%)**	Depression (14.6) Drug abuse (3.2) Anxiety (2.6)	Depression (38.7) Anxiety (9.4)	Depression (51.0) Anxiety (12.9) Drug abuse (5.8)	Depression (50.5) Anxiety (15.3)	Depression (22.7) Drug abuse (4.1) Anxiety (3.4)	Depression (43.4) Anxiety (7.6) Obsessive-compulsive disorder (2.7)	Depression (38.1) Anxiety (14.8) Panic disorder/attack (6.5)	Depression (32.8) Anxiety (9.9) Generalised anxiety disorder (3.9)	Depression (34.5) Anxiety (7.5)	Depression (44.8) Anxiety (15.1)
**ROA (%)**	Oral (68.4) Parenteral * (0.8) T-placent (0.7) Inhalation (0.2) NA (29.9)	Oral (70.7) T-placent (8.3) Parenteral * (0.3) T-mam (0.2) NA (20.4)	Oral (79.7) T-placent (2.5) NA (16.6)	Oral (77.8) T-placent (6.3) T-mam (0.2) NA: (15.7)	Oral (61.9) Inhalation (1.9) T-placent (1.7) Parenteral * (1.2) NA (33.2)	Oral (57.4) T-placent (15.0) T-mam (0.3) Parenteral * (0.2) NA (27.2)	Oral (82.2) T-placent (1.4) Parenteral * (0.2) Inhalation (0.1) NA (16.0)	Oral (74.7) T-placent (10.3) Parenteral * (0.1) NA (15.1)	Oral (65.5) T-placent (2.2) Inhalation (0.5) Parenteral * (0.4) NA (30.9)	Oral (69.4) T-placent (11.7) T-mam (0.3) NA (15.7)
**Therapeutic regimen (Mono/Poly)**	139 (10%)/ 1280 (90%)	1502 (6%)/ 24,242 (94%)	131 (32%)/ 273 (68%)	1260 (7%)/ 16,975 (93%)	76 (10%)/ 695 (90%)	1072 (5%)/ 21,721 (95%)	537 (34%)/ 1055 (66%)	3586 (9%)/ 35,505 (91%)	197 (17%)/ 952 (83%)	3365 (9%)/ 35,167 (91%)
**Most important concomitant prescription psychotropic drugs recorded (%)**										
Antidepressants	17.5	19.2	10.4	17.3	18.8	20.2	9.0	11.0	13.4	13.6
Antihistamines	25.4	9.0	3.2	5.7	19.3	8.5	10.8	4.6	17.5	6.5
Antipsychotics	13.7	17.1	16.1	19.0	19.3	18.7	9.5	9.8	13.2	15.8
Benzodiazepines °	36.9	22.8	28.7	23.8	43.3	20.4	27.6	17.2	29.9	16.4
Gabapentinoids	2.5	5.0	4.5	4.2	4.2	4.8	2.3	2.0	3.7	4.7
Mood Stabilizers	3.0	8.4	7.2	10.3	5.4	9.2	3.7	5.4	7.0	7.0
Opioids	59.7	22.7	10.9	11.0	43.3	16.4	16.0	7.1	40.7	11.1
Z-Drugs	3.4	6.3	4.5	6.8	9.3	5.6	8.2	4.8	5.6	4.5
**Most important concomitant recreational drugs recorded (%)**										
Alcohol	13.5	4.3	2.7	1.7	14.8	3.1	3.5	1.4	7.1	1.3
Amphetamines	2.4	1.1	1.5	0.7	3.1	1.2	0.5	0.3	3.3	0.7
Cannabis and Cannabinoids	1.2	0.6	1.7	0.4	1.3	0.4	0.5	0.2	1.2	0.3
Cocaine	9.4	1.5	2.2	0.4	4.9	0.6	1.0	0.2	9.8	0.6
Heroin	0	1.8	0	0	0	0.5	0	0.1	0	0.5
Ketamine	0.2	0	0	0	0.4	0	0	0	0	0

Abbreviations: EMA: European Medicines Agency; FAERS: Food and Drug Administration Adverse Event Reporting System; Mono: monotherapy; NA: not available; Poly: polytherapy; ROA: route of administration; SD: Standard Deviation; SSRI: Selective Serotonin Reuptake Inhibitors; T-mam: Transmammary; T-placent: Transplacental; * Parenteral refers to intramuscular, subcutaneous, and intravenous administrations; ° excluding Z-drugs.

**Table 2:** Outcome of selective serotonin reuptake inhibitor (SSRI)-related adverse drug reactions reported to the European Medicines Agency (EMA) EudraVigilance (EV) dataset and the Food and Drug Administration (FDA) Adverse Event Reporting System.

EMA			FAERS		
Drug (Total Cases)	Cases with Fatal Outcome Sex ° (%) and Mean Age (SD)	Percent of Drug-Specific Cases *	Drug (Total Cases)	Cases with Fatal Outcome Sex ° (%) and Mean Age (SD)	Percent of Drug- Specific Cases
**Citalopram (1419)**	994 F: 49.6% Mean age: 42.0 yy (12.4)	70.0%	**Citalopram (25,744)**	7402 F: 50.0% Mean age: 45.7 yy (17.1)	28.8%
**Escitalopram (404)**	31 F: 54.8% Mean age: 40.4 yy (16.0)	7.7%	**Escitalopram (18,235)**	2293 F: 50.6% Mean age: 48.3 yy (20.7)	12.6%
**Fluoxetine (771)**	424 F: 55.7% Mean age: 44.3 yy (12.9)	55.0%	**Fluoxetine (22,793)**	4659 F: 53.8% Mean age: 44.1 yy (17.5)	20.4%
**Paroxetine (1592)**	271 F: 44.6% Mean age: 43.6 yy (12.0)	17.0%	**Paroxetine (39,091)**	3438 F: 45.2% Mean age: 48.8 yy (20.9)	8.8%
**Sertraline (1149)**	532 F: 41.0% Mean age: 41.8 yy (14.0)	46.3%	**Sertraline (38,532)**	4863 F: 45.2% Mean age 48.1 yy (20.8)	12.6%

EMA: European Medicines Agency; F: female; FAERS: Food and Drug Administration Adverse Event Reporting System; SD: standard deviation: yy: years; ° the female rate is reported; * % of observations where the index SSRI was the only drug suspected.

## Data Availability

Restrictions apply to the availability of the EudraVigilance data. Data was obtained and are available by request from the European Medicines Agency. The FDA Adverse Event Reporting System data are publicly available and can be found here: https://www.fda.gov/drugs/questions-and-answers-fdas-adverse-event-reporting-system-faers/fda-adverse-event-reporting-system-faers-public-dashboard (accessed on 28 February 2022).

## Supplementary Materials

The following supporting information can be downloaded at: https://www.mdpi.com/article/10.3390/ph15050565/s1, Table S1: Most common countries of origin and adverse events reported in SSRI-related adverse drug reaction reports recorded in the European Medicines Agency (EMA) EudraVigilance (EV) dataset and the Food and Drug Administration (FDA) Adverse Event Reporting System (FAERS); Table S2: Signal scores regarding abuse/dependence and withdrawal issues for citalopram, escitalopram, fluoxetine, paroxetine, and sertraline (European Medicines Agency/EMA and the Food and Drug Administration—FDA Adverse Event Reporting System/FAERS datasets); Table S3: Other signal scores for citalopram, escitalopram, fluoxetine, paroxetine, and sertraline (European Medicines Agency/EMA and the Food and Drug Administration Adverse Event Reporting System/FAERS datasets); Table S4: Estimated US prevalence of prescription antidepressant use in past month according to the National Health and Nutrition Examination Survey (NHANES) data (2003–2018); Figure S1: Number of annual prescriptions of selected SSRIs from Prescription Cost Analysis (England) data (2004–2018).
