# Supplementary material for: A Focus on Abuse/Misuse and Withdrawal Issues with Selective Serotonin Reuptake Inhibitors (SSRIs): Analysis of Both the European EMA and the US FAERS Pharmacovigilance Databases
Source: Pharmaceuticals (Basel). 2022 May 1;15(5):565. doi: 10.3390/ph15050565 (PMC9146999; doi:10.3390/ph15050565)
Supplement: Supplementary file 1 [file pharmaceuticals-15-00565-s001.zip › Figure S1_R3.pdf]

Figure S1. Number of annual prescriptions of selected SSRIs from Prescription Cost Analysis (England) data (2004-2018).

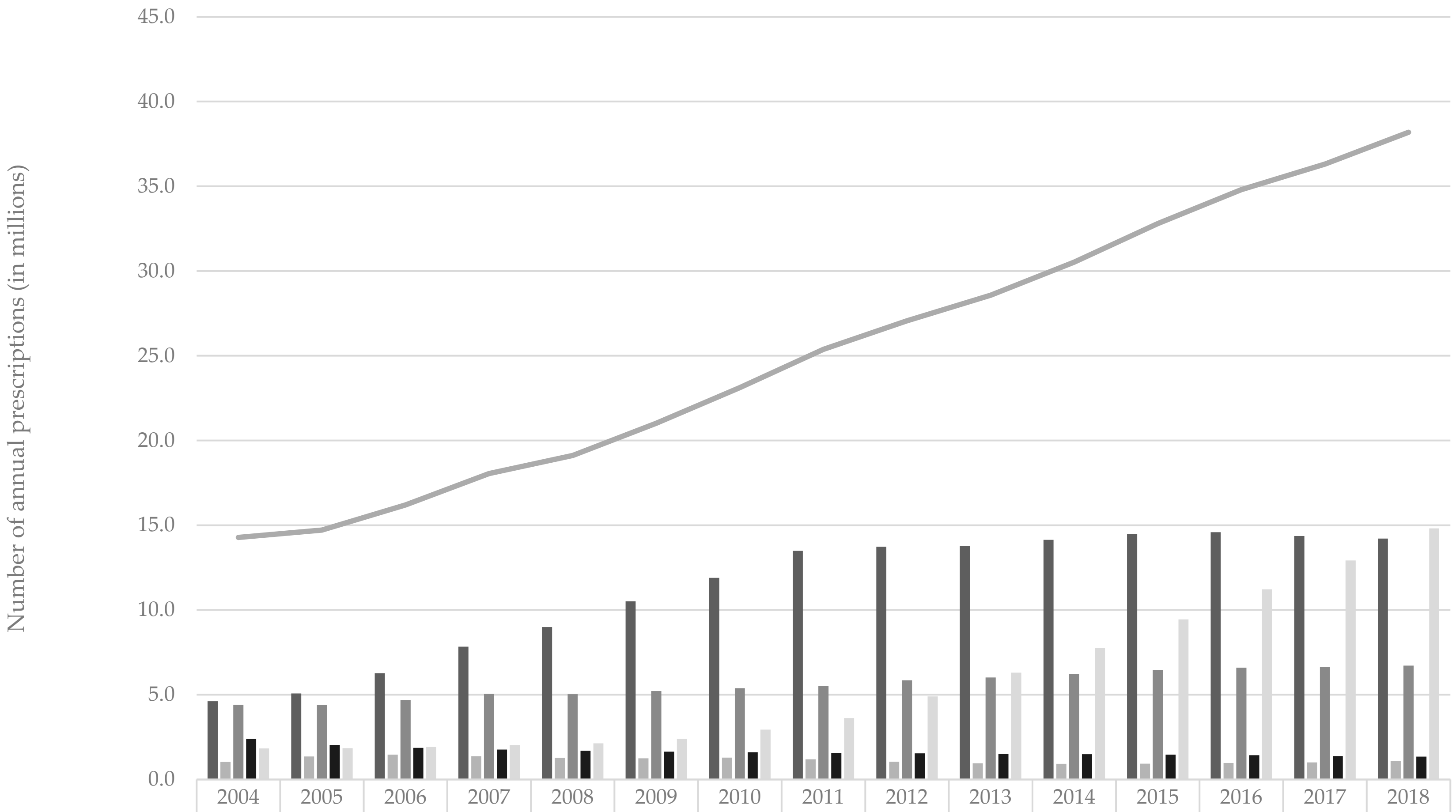

	2004	2005	2006	2007	2008	2009	2010	2011	2012	2013	2014	2015	2016	2017	2018
Citalopram	4.6	5.1	6.3	7.8	9.0	10.5	11.9	13.5	13.7	13.8	14.1	14.5	14.6	14.4	14.2
Escitalopram	1.0	1.4	1.5	1.4	1.3	1.3	1.3	1.2	1.0	1.0	0.9	0.9	1.0	1.0	1.1
Fluoxetine	4.4	4.4	4.7	5.0	5.0	5.2	5.4	5.5	5.8	6.0	6.2	6.5	6.6	6.6	6.7
Paroxetine	2.4	2.0	1.9	1.8	1.7	1.6	1.6	1.6	1.5	1.5	1.5	1.5	1.4	1.4	1.4
Sertraline	1.8	1.8	1.9	2.0	2.1	2.4	2.9	3.6	4.9	6.3	7.8	9.4	11.2	12.9	14.8
Total	14.3	14.7	16.2	18.1	19.1	21.0	23.1	25.4	27.1	28.6	30.5	32.8	34.8	36.3	38.2

Note: Prescription item is defined as a single item prescribed by a doctor, dentist, or nurse on a prescription form.
