# Supplementary material for: A Focus on Abuse/Misuse and Withdrawal Issues with Selective Serotonin Reuptake Inhibitors (SSRIs): Analysis of Both the European EMA and the US FAERS Pharmacovigilance Databases
Source: Pharmaceuticals (Basel). 2022 May 1;15(5):565. doi: 10.3390/ph15050565 (PMC9146999; doi:10.3390/ph15050565)
Supplement: Supplementary file 1 [file pharmaceuticals-15-00565-s001.zip › TableS1_R3.pdf]

Table S1. Most common countries of origin and adverse events reported in SSRI-related adverse drug reaction reports recorded in the European Medicines Agency (EMA) EudraVigilance (EV) dataset and the Food and Drug Administration (FDA) Adverse Event Reporting System (FAERS).

	CITALOPRAM		ESCITALOPRAM		FLUOXETINE		PAROXETINE		SERTRALINE	
	**EMA**	**FAERS**	**EMA**	**FAERS**	**EMA**	**FAERS**	**EMA**	**FAERS**	**EMA**	**FAERS**
**Country of origin (most recorded countries, %)**	US (73.1)<br>Italy (7.1)<br>Germany (6.3)<br>Canada (4.3)<br>UK (2.9)	US (37.7)<br>UK (25.3)<br>Germany (10.1)<br>Italy (6.7)<br>France (4.1)	US (31.4)<br>Italy (16.4)<br>Germany (8.8)<br>France (8.2)<br>Ireland (4.1)	US (39.2)<br>France (20.1)<br>Italy (5.7)<br>Germany (4.9)<br>Switzerland (4.0)	US (70.2)<br>Italy (6.8)<br>Germany (3.9)<br>France (3.0)<br>Switzerland (2.1)	US (55.1)<br>UK (15.5)<br>France (7.2)<br>Germany (3.3)<br>Italy (2.4)	US (56.7)<br>Japan (11.0)<br>UK (7.6)<br>Canada (6.2)<br>Netherlands (3.2)	US (66.1)<br>Japan (7.7)<br>France (7.3)<br>UK (6.5)<br>Italy (2.7)	US (67.9)<br>Italy (5.3)<br>Germany (5.2)<br>UK (2.6)<br>Japan (2.3)	US (50.6)<br>UK (19.1)<br>Germany (4.7)<br>Italy (4.0)<br>Japan (3.4)
**Most common adverse events reported (n of ADR)**	Drug abuse (937)<br><br>Toxicity to various agents (362)<br><br>Intentional product misuse (216)<br><br>Withdrawal syndrome/Drug withdrawal syndrome (244)<br><br>Cardiac/Respiratory/Cardio-respiratory arrest (207)<br><br>Death (124)<br><br>Anxiety (52)	Toxicity to various agents (2,708)<br><br>Completed suicide (2,687)<br><br>Drug interaction (2,191)<br><br>Drug abuse (1,773)<br><br>Overdose (1,309)<br><br>Anxiety (1,040)<br><br>Dizziness (959)<br><br>Nausea (936)<br><br>Serotonin syndrome (935)<br><br>Intentional overdose (850)	Withdrawal syndrome/Drug withdrawal syndrome (249)<br><br>Drug abuse (92)<br><br>Intentional product misuse (60)<br><br>Dizziness (53)<br><br>Anxiety (43)<br><br>Suicidal ideation (38)<br><br>Nausea (37)<br><br>Headache (35)<br><br>Depression (30)	Drug interaction (1,377)<br><br>Completed suicide (1,245)<br><br>Anxiety (1,003)<br><br>Depression (944)<br><br>Nausea (935)<br><br>Suicidal ideation (840)<br><br>Dizziness (837)<br><br>Drug ineffective (797)<br><br>Headache (722)<br><br>Overdose (714)	Drug abuse (450)<br><br>Withdrawal syndrome/Drug withdrawal syndrome (203)<br><br>Intentional product misuse (122)<br><br>Death (121)<br><br>Toxicity to various agents (112)<br><br>Suicide attempt (45)<br><br>Suicidal ideation (44)<br>Anxiety (43)<br><br>Cardio-respiratory arrest (38)	Completed suicide (2,052)<br><br>Drug interaction (1,757)<br><br>Foetal exposure (1,572)<br><br>Toxicity to various agents (1,501)<br><br>Drug ineffective (1,232)<br><br>Depression (1,169)<br><br>Anxiety (1,027)<br><br>Maternal exposure during pregnancy (1,024)<br><br>Suicidal ideation (907)<br><br>Drug abuse (798)	Withdrawal syndrome/Drug withdrawal syndrome (1,267)<br><br>Dizziness (424)<br><br>Nausea (396)<br><br>Anxiety (383)<br><br>Paraesthesia (341)<br><br>Suicidal ideation (341)<br><br>Headache (293)<br><br>Tremor (271)<br><br>Insomnia (259)	Drug withdrawal syndrome (6,858)<br><br>Dizziness (3,517)<br><br>Maternal exposure during pregnancy (3,503)<br><br>Anxiety (3,073)<br><br>Nausea (3,044)<br><br>Drug ineffective (2,632)<br><br>Suicidal ideation (2,497)<br><br>Headache (2,319)<br><br>Insomnia (2,182)<br><br>Foetal exposure (2,161)	Drug abuse (601)<br><br>Withdrawal syndrome/Drug withdrawal syndrome (401)<br><br>Toxicity to various agents (160)<br><br>Intentional product misuse (130)<br><br>Anxiety (88)<br><br>Dizziness (77)<br><br>Depression (69)<br><br>Drug ineffective (67)<br><br>Nausea (64)	Drug ineffective (2,631)<br><br>Anxiety (2,299)<br><br>Drug interaction (1,970)<br><br>Depression (1,952)<br><br>Nausea (1,782)<br><br>Insomnia (1,734)<br><br>Completed suicide (1,672)<br><br>Dizziness (1,644)<br><br>Headache (1,561)<br><br>Fatigue (1,547)

Abbreviations: ADR: adverse drug reaction; EMA: European Medicines Agency; FAERS: FDA Adverse Event Reporting System; SSRI: selective serotonin reuptake inhibitor; UK: United Kingdom; US: United States
