# Supplementary material for: A Focus on Abuse/Misuse and Withdrawal Issues with Selective Serotonin Reuptake Inhibitors (SSRIs): Analysis of Both the European EMA and the US FAERS Pharmacovigilance Databases
Source: Pharmaceuticals (Basel). 2022 May 1;15(5):565. doi: 10.3390/ph15050565 (PMC9146999; doi:10.3390/ph15050565)
Supplement: Supplementary file 1 [file pharmaceuticals-15-00565-s001.zip › TableS2_R3.pdf]

**Table S2. Signal scores regarding abuse/dependence and withdrawal issues for citalopram, escitalopram, fluoxetine, paroxetine, and sertraline (European Medicines Agency/EMA and the Food and Drug Administration-FDA Adverse Event Reporting System/FAERS datasets).**

| Preferred terms (PT) | Citalopram | | | | Escitalopram | | | | Fluoxetine | | | | Paroxetine | | | | Sertraline | | | |
|---|---|---|---|---|---|---|---|---|---|---|---|---|---|---|---|---|---|---|---|---|
| | PRR | ROR | IC025 | EB05 | PRR | ROR | IC025 | EB05 | PRR | ROR | IC025 | EB05 | PRR | ROR | IC025 | EB05 | PRR | ROR | IC025 | EB05 |
| **Misuse/abuse-related terms** | | | | | | | | | | | | | | | | | | | | |
| EMA- Drug abuse | **4.12 (<0.01)** | **5.00 (<0.01)** | **1.39 (<0.01)** | **2.69 (<0.01)** | 0.48 (0.42) | 0.46 (0.42) | -1.31 (0.30) | 0.42 (0.29) | **1.77 (<0.01)** | **1.88 (<0.01)** | **0.54 (<0.01)** | **1.50 (<0.01)** | 0.12 (0.43) | 0.10 (0.43) | -2.49 (0.42) | 0.18 (0.41) | **1.57 (<0.01)** | **1.64 (<0.01)** | **0.37 (<0.01)** | **1.33 (<0.01)** |
| FAERS- Drug abuse | **3.35 (<0.01)** | **3.39 (<0.01)** | **1.18 (<0.01)** | **2.31 (<0.01)** | 0.52 (0.63) | 0.51 (0.63) | -1.04 (0.27) | 0.50 (0.27) | **1.22 (<0.01)** | **1.22 (<0.01)** | **0.13 (<0.01)** | **1.11 (0.01)** | 0.32 (0.63) | 0.32 (0.63) | -1.44 (0.33) | 0.38 (0.34) | 0.86 (0.63) | 0.86 (0.63) | -0.27 (0.06) | 0.85 (0.07) |
| EMA- Drug abuser | **3.00 (0.03)** | **3.00 (0.03)** | **-2.58 (0.42)** | 0.37 (0.32) | **5.74 (<0.01)** | **5.75 (<0.01)** | **-2.33 (0.41)** | 0.41 (0.30) | NA | NA | NA | NA | NA | NA | NA | NA | 2.22 (0.08) | 2.22 (0.08) | -2.72 (0.43) | 0.35 (0.33) |
| FAERS- Drug abuser | **1.32 (0.01)** | **1.32 (0.01)** | **-0.18 (0.03)** | **0.95 (0.02)** | 0.74 (0.56) | 0.74 (0.56) | -1.08 (0.28) | 0.52 (0.26) | **1.46 (<0.01)** | **1.46 (<0.01)** | **-0.06 (0.01)** | **1.03 (0.01)** | 0.70 (0.60) | 0.70 (0.60) | -0.86 (0.23) | 0.60 (0.22) | 1.01 (0.35) | 1.01 (0.35) | -0.45 (0.12) | 0.79 (0.10) |
| EMA- Drug diversion | NA | NA | NA | NA | **5.74 (<0.01)** | **5.75 (<0.01)** | **-1.30 (0.30)** | 0.61 (0.17) | NA | NA | NA | NA | 0.56 (0.31) | 0.56 (0.31) | -2.77 (0.43) | 0.29 (0.36) | **2.22 (0.02)** | **2.22 (0.02)** | -1.85 (0.37) | 0.48 (0.26) |
| FAERS- Drug diversion | 0.19 (0.58) | 0.19 (0.58) | -4.20 (0.49) | 0.11 (0.47) | 1.61 (0.11) | 1.61 (0.11) | -0.99 (0.26) | 0.62 (0.21) | 0.69 (0.49) | 0.69 (0.49) | -2.16 (0.40) | 0.31 (0.38) | 0.41 (0.58) | 0.41 (0.58) | -2.47 (0.43) | 0.25 (0.41) | **3.11 (<0.01)** | **3.11 (<0.01)** | **-0.07 (0.01)** | **1.14 (<0.01)** |
| EMA- Drug use disorder | NA | NA | NA | NA | **11.49 (<0.01)** | **11.49 (<0.01)** | -2.33 (0.41) | 0.43 (0.29) | **7.30 (<0.01)** | **7.30 (<0.01)** | -2.47 (0.42) | 0.41 (0.30) | NA | NA | NA | NA | NA | NA | NA | NA |
| FAERS- Drug use disorder | 0.18 (0.58) | 0.18 (0.58) | -4.24 (0.49) | 0.11 (0.47) | **1.93 (0.01)** | **1.93 (0.01)** | -0.70 (0.20) | 0.74 (0.13) | **9.96 (<0.01)** | **9.96 (<0.01)** | **0.95 (<0.01)** | **2.19 (<0.01)** | 0.09 (0.61) | 0.09 (0.61) | -4.97 (0.50) | 0.07 (0.47) | 0.22 (0.60) | 0.22 (0.60) | -3.58 (0.48) | 0.14 (0.46) |
| EMA- Intentional product misuse | **3.20 (<0.01)** | **3.32 (<0.01)** | **1.05 (<0.01)** | **2.15 (<0.01)** | **1.23 (<0.01)** | **1.23 (<0.01)** | **-0.13 (0.02)** | **0.96 (0.02)** | **1.78 (<0.01)** | **1.81 (<0.01)** | **0.41 (<0.01)** | **1.39 (<0.01)** | 0.20 (0.42) | 0.19 (0.42) | -1.97 (0.38) | 0.27 (0.37) | **1.18 (<0.01)** | **1.18 (<0.01)** | **-0.09 (0.01)** | **0.98 (0.02)** |
| FAERS- Intentional product misuse | **2.22 (<0.01)** | **2.23 (<0.01)** | **0.71 (<0.01)** | **1.68 (<0.01)** | 0.86 (0.58) | 0.86 (0.58) | -0.46 (0.13) | 0.75 (0.12) | **1.43 (<0.01)** | **1.43 (<0.01)** | **0.22 (<0.01)** | **1.20 (<0.01)** | 0.45 (0.63) | 0.45 (0.63) | -1.12 (0.28) | 0.48 (0.28) | 0.80 (0.62) | 0.80 (0.62) | -0.44 (0.12) | 0.76 (0.12) |
| EMA- Substance abuse | **3.85 (<0.01)** | **3.88 (<0.01)** | **0.87 (<0.01)** | **1.99 (<0.01)** | 0.66 (0.39) | 0.66 (0.39) | -1.74 (0.36) | 0.37 (0.31) | **1.24 (0.04)** | **1.24 (0.04)** | -0.54 (0.09) | 0.78 (0.08) | 0.23 (0.42) | 0.23 (0.42) | -2.14 (0.40) | 0.26 (0.38) | **1.38 (<0.01)** | **1.38 (<0.01)** | **-0.30 (0.05)** | **0.91 (0.03)** |
| FAERS- Substance abuse | **1.83 (<0.01)** | **1.83 (<0.01)** | **0.11 (<0.01)** | **1.17 (<0.01)** | 0.39 (0.61) | 0.39 (0.61) | -2.29 (0.41) | 0.25 (0.41) | 0.80 (0.52) | 0.80 (0.52) | -1.02 (0.26) | 0.55 (0.24) | 0.89 (0.48) | 0.89 (0.48) | -0.66 (0.19) | 0.70 (0.16) | 1.10 (0.23) | 1.10 (0.23) | -0.45 (0.12) | 0.80 (0.09) |
| EMA- Substance use | **3.00 (0.03)** | **3.00 (0.03)** | -2.58 (0.42) | 0.37 (0.32) | NA | NA | NA | NA | **3.65 (<0.01)** | **3.65 (<0.01)** | -2.50 (0.42) | 0.38 (0.31) | NA | NA | NA | NA | 2.22 (0.09) | 2.22 (0.09) | -2.72 (0.43) | 0.35 (0.33) |
| FAERS- Substance use | **1.90 (0.01)** | **1.90 (0.01)** | -0.58 (0.16) | 0.80 (0.09) | 0.25 (0.56) | 0.25 (0.56) | -3.93 (0.49) | 0.13 (0.46) | 1.44 (0.15) | 1.44 (0.15) | -0.98 (0.26) | 0.62 (0.21) | 0.08 (0.61) | 0.08 (0.61) | -5.01 (0.50) | 0.07 (0.48) | **2.35 (<0.01)** | **2.35 (<0.01)** | -0.27 (0.06) | **0.99 (0.01)** |
| **Dependence-related terms** | | | | | | | | | | | | | | | | | | | | |
| EMA- Dependence | 0.16 (0.42) | 0.16 (0.42) | -3.58 (0.47) | 0.11 (0.42) | 0.31 (0.40) | 0.31 (0.40) | -2.78 (0.43) | 0.19 (0.40) | 0.28 (0.41) | 0.28 (0.41) | -2.73 (0.43) | 0.19 (0.40) | **6.45 (<0.01)** | **6.51 (<0.01)** | **0.53 (<0.01)** | **1.57 (<0.01)** | 0.27 (0.42) | 0.27 (0.42) | -2.50 (0.42) | 0.21 (0.40) |

| | | | | | | | | | | | | | | | | | | | | |
|---|---|---|---|---|---|---|---|---|---|---|---|---|---|---|---|---|---|---|---|---|
| **FAERS-Dependence** | 0.07 (0.63) | 0.07 (0.63) | -4.45 (0.50) | 0.05 (0.48) | 0.06 (0.63) | 0.06 (0.63) | -5.09 (0.50) | 0.04 (0.48) | 0.11 (0.63) | 0.11 (0.63) | -3.67 (0.48) | 0.09 (0.47) | **27.42 (<0.01)** | **27.51 (<0.01)** | **1.46 (<0.01)** | **2.86 (<0.01)** | 0.11 (0.63) | 0.11 (0.63) | -3.36 (0.47) | 0.11 (0.47) |
| **EMA- Drug dependence** | 0.41 (0.41) | 0.41 (0.41) | -1.89 (0.37) | 0.31 (0.35) | 1.02 (0.19) | 1.02 (0.19) | -0.68 (0.14) | 0.69 (0.13) | 0.57 (0.39) | 0.57 (0.39) | -1.44 (0.32) | 0.42 (0.30) | **1.84 (<0.01)** | **1.84 (<0.01)** | **0.09 (<0.01)** | **1.14 (0.01)** | 0.84 (0.39) | 0.84 (0.39) | -0.73 (0.16) | 0.66 (0.15) |
| **FAERS-Drug dependence** | 0.33 (0.63) | 0.33 (0.63) | -1.77 (0.37) | 0.31 (0.38) | 0.44 (0.63) | 0.44 (0.63) | -1.43 (0.33) | 0.39 (0.33) | 0.34 (0.63) | 0.34 (0.63) | -1.73 (0.36) | 0.32 (0.37) | **3.61 (<0.01)** | **3.62 (<0.01)** | **0.88 (<0.01)** | **1.90 (<0.01)** | 0.79 (0.62) | 0.79 (0.62) | -0.45 (0.12) | 0.75 (0.12) |
| **EMA-Substance dependence** | NA | NA | NA | NA | NA | NA | NA | NA | NA | NA | NA | NA | 0.56 (0.28) | 0.56 (0.28) | -3.46 (0.47) | 0.25 (0.38) | **8.89 (<0.01)** | **8.89 (<0.01)** | -1.61 (0.34) | 0.59 (0.18) |
| **FAERS-Substance dependence** | NA | NA | NA | NA | NA | NA | NA | NA | 1.84 (0.15) | 1.84 (0.15) | -1.86 (0.37) | 0.42 (0.32) | 1.41 (0.25) | 1.41 (0.25) | -1.78 (0.37) | 0.44 (0.30) | 1.73 (0.15) | 1.73 (0.15) | -1.63 (0.35) | 0.48 (0.28) |
| **Withdrawal-related terms** | | | | | | | | | | | | | | | | | | | | |
| **EMA- Drug withdrawal syndrome** | 1.01 (0.19) | 1.01 (0.19) | **-0.29 (0.05)** | 0.86 (0.05) | **1.68 (<0.01)** | **1.71 (<0.01)** | **0.34 (<0.01)** | **1.32 (<0.01)** | 0.90 (0.36) | 0.90 (0.36) | -0.48 (0.08) | 0.76 (0.09) | 1.01 (0.19) | 1.01 (0.19) | **-0.18 (0.02)** | **0.92 (0.03)** | 0.75 (0.40) | 0.74 (0.40) | -0.65 (0.13) | 0.67 (0.14) |
| **FAERS-Drug withdrawal syndrome** | 0.13 (0.63) | 0.13 (0.63) | -2.89 (0.45) | 0.14 (0.46) | 0.17 (0.63) | 0.17 (0.63) | -2.57 (0.43) | 0.17 (0.45) | 0.19 (0.63) | 0.19 (0.63) | -2.37 (0.42) | 0.20 (0.43) | **13.68 (<0.01)** | **14.19 (<0.01)** | **1.47 (<0.01)** | **2.80 (<0.01)** | 0.19 (0.63) | 0.19 (0.63) | -2.16 (0.40) | 0.23 (0.42) |
| **Overdose and off label use terms** | | | | | | | | | | | | | | | | | | | | |
| **EMA-Intentional overdose** | **1.56 (<0.01)** | **1.57 (<0.01)** | **-0.25 (0.04)** | **0.95 (0.02)** | 0.80 (0.30) | 0.80 (0.30) | -1.52 (0.33) | 0.43 (0.29) | **2.58 (<0.01)** | **2.59 (<0.01)** | **0.37 (<0.01)** | **1.42 (<0.01)** | 0.31 (0.42) | 0.31 (0.42) | -1.82 (0.36) | 0.33 (0.34) | **1.48 (<0.01)** | **1.48 (<0.01)** | **-0.27 (0.04)** | **0.93 (0.03)** |
| **FAERS-Intentional overdose** | **1.65 (<0.01)** | **1.65 (<0.01)** | **0.46 (<0.01)** | **1.40 (<0.01)** | **1.59 (<0.01)** | **1.59 (<0.01)** | **0.44 (<0.01)** | **1.38 (<0.01)** | **1.30 (<0.01)** | **1.30 (<0.01)** | **0.19 (<0.01)** | **1.17 (<0.01)** | 0.50 (0.63) | 0.50 (0.63) | -0.90 (0.24) | 0.55 (0.25) | 0.74 (0.63) | 0.74 (0.63) | -0.46 (0.12) | 0.74 (0.13) |
| **EMA- off-label use** | 1.09 (0.16) | 1.09 (0.16) | -1.22 (0.29) | 0.54 (0.22) | **1.69 (<0.01)** | **1.69 (<0.01)** | -0.88 (0.19) | 0.67 (0.14) | **3.24 (<0.01)** | **3.25 (<0.01)** | **0.20 (<0.01)** | **1.32 (<0.01)** | 0.38 (0.40) | 0.38 (0.40) | -1.89 (0.37) | 0.34 (0.34) | 0.81 (0.28) | 0.81 (0.28) | -1.54 (0.33) | 0.44 (0.28) |
| **FAERS- off-label use** | **1.14 (<0.01)** | **1.14 (<0.01)** | **-0.02 (0.01)** | **1.01 (0.01)** | **2.13 (<0.01)** | **2.14 (<0.01)** | **0.73 (<0.01)** | **1.71 (<0.01)** | **2.00 (<0.01)** | **2.00 (<0.01)** | **0.63 (<0.01)** | **1.59 (<0.01)** | 0.36 (0.63) | 0.36 (0.63) | -1.38 (0.32) | 0.40 (0.33) | 0.65 (0.63) | 0.65 (0.63) | -0.66 (0.19) | 0.65 (0.19) |
| **EMA-Overdose** | **1.53 (<0.01)** | **1.54 (<0.01)** | **-0.02 (0.01)** | **1.07 (0.01)** | **1.26 (0.01)** | **1.26 (0.01)** | -0.42 (0.07) | 0.82 (0.06) | **1.35 (<0.01)** | **1.35 (<0.01)** | **-0.22 (0.03)** | **0.94 (0.03)** | 0.40 (0.42) | 0.40 (0.42) | -1.28 (0.30) | 0.45 (0.28) | **1.69 (<0.01)** | **1.70 (<0.01)** | **0.12 (<0.01)** | **1.17 (<0.01)** |
| **FAERS-Overdose** | **1.88 (<0.01)** | **1.89 (<0.01)** | **0.62 (<0.01)** | **1.56 (<0.01)** | **1.25 (<0.01)** | **1.25 (<0.01)** | **0.16 (<0.01)** | **1.14 (<0.01)** | **1.06 (0.01)** | **1.06 (0.01)** | **-0.04 (0.01)** | **0.99 (0.01)** | 0.62 (0.63) | 0.62 (0.63) | -0.61 (0.17) | 0.67 (0.18) | 0.75 (0.63) | 0.75 (0.63) | -0.42 (0.11) | 0.76 (0.12) |

Boldface denotes signals based on FDR<0.05; Minimum number of events to compute signal statistics = 5 for all measures.

Abbreviations: EMA: European Medicines Agency; EB05 = 5% quantile of the posterior distribution of the empirical Bayesian geometric mean (estimated FDR); FAERS: Food and Drug Administration Adverse Event Reporting System; FDR = false discovery rate; IC025= 2.5% quantile of the posterior distribution of information component (estimated FDR); NA = not available = less than 5 events for this pair; PRR= observed relative risks (estimated FDR); ROR= observed odds ratios (estimated FDR).
