# Supplementary material for: A Focus on Abuse/Misuse and Withdrawal Issues with Selective Serotonin Reuptake Inhibitors (SSRIs): Analysis of Both the European EMA and the US FAERS Pharmacovigilance Databases
Source: Pharmaceuticals (Basel). 2022 May 1;15(5):565. doi: 10.3390/ph15050565 (PMC9146999; doi:10.3390/ph15050565)
Supplement: Supplementary file 1 [file pharmaceuticals-15-00565-s001.zip › TableS3_R3.pdf]

**Table S3. Other signal scores for citalopram, escitalopram, fluoxetine, paroxetine, and sertraline (European Medicines Agency/EMA and the Food and Drug Administration Adverse Event Reporting System/ FAERS datasets).**

| Preferred term (PT) | Citalopram | | | | Escitalopram | | | | Fluoxetine | | | | Paroxetine | | | | Sertraline | | | |
|---|---|---|---|---|---|---|---|---|---|---|---|---|---|---|---|---|---|---|---|---|
| | PRR | ROR | IC025 | EB05 | PRR | ROR | IC025 | EB05 | PRR | ROR | IC025 | EB05 | PRR | ROR | IC025 | EB05 | PRR | ROR | IC025 | EB05 |
| **EMA-Acute psychosis** | NA | NA | NA | NA | NA | NA | NA | NA | NA | NA | NA | NA | Inf (0.48) | Inf (0.48) | -3.17 (0.46) | 0.36 (0.32) | NA | NA | NA | NA |
| **FAERS-Acute psychosis** | **1.71 (<0.01)** | **1.71 (<0.01)** | **-0.13 (0.02)** | **1.02 (0.01)** | **1.62 (<0.01)** | **1.62 (<0.01)** | -0.26 (0.06) | **0.93 (0.03)** | 0.99 (0.36) | 0.99 (0.36) | -0.90 (0.24) | 0.61 (0.21) | 0.14 (0.62) | 0.14 (0.62) | -3.53 (0.48) | 0.11 (0.47) | **1.55 (<0.01)** | **1.55 (<0.01)** | **-0.20 (0.04)** | **0.97 (0.02)** |
| **EMA-Aggression** | 0.25 (0.42) | 0.25 (0.42) | -2.71 (0.43) | 0.18 (0.41) | 0.52 (0.39) | 0.52 (0.39) | -1.74 (0.36) | 0.35 (0.33) | 0.30 (0.42) | 0.30 (0.42) | -2.47 (0.42) | 0.21 (0.40) | **3.02 (<0.01)** | **3.04 (<0.01)** | **0.34 (<0.01)** | **1.36 (<0.01)** | 0.76 (0.40) | 0.76 (0.40) | -0.83 (0.18) | 0.61 (0.17) |
| **FAERS-Aggression** | 0.56 (0.63) | 0.56 (0.63) | -0.89 (0.24) | 0.55 (0.24) | 0.65 (0.63) | 0.64 (0.63) | -0.74 (0.21) | 0.61 (0.21) | 0.75 (0.63) | 0.75 (0.63) | -0.51 (0.14) | 0.72 (0.14) | **2.45 (<0.01)** | **2.46 (<0.01)** | **0.69 (<0.01)** | **1.64 (<0.01)** | 0.66 (0.63) | 0.66 (0.63) | -0.60 (0.17) | 0.67 (0.17) |
| **EMA-Ataxia** | NA | NA | NA | NA | 0.50 (0.32) | 0.50 (0.32) | -3.29 (0.46) | 0.22 (0.39) | **2.43 (<0.01)** | **2.43 (<0.01)** | -0.52 (0.09) | **0.86 (0.05)** | **2.23 (<0.01)** | **2.23 (<0.01)** | -0.51 (0.09) | **0.89 (0.04)** | NA | NA | NA | NA |
| **FAERS-Ataxia** | **2.31 (<0.01)** | **2.31 (<0.01)** | **0.46 (<0.01)** | **1.47 (<0.01)** | 0.89 (0.47) | 0.89 (0.47) | -0.84 (0.23) | 0.61 (0.21) | 0.81 (0.53) | 0.81 (0.53) | -0.90 (0.24) | 0.59 (0.22) | 0.47 (0.62) | 0.47 (0.62) | -1.40 (0.33) | 0.42 (0.32) | 1.11 (0.19) | 1.11 (0.19) | -0.36 (0.09) | 0.84 (0.07) |
| **EMA-Confusional state** | 0.38 (0.42) | 0.38 (0.42) | -1.91 (0.37) | 0.30 (0.36) | 0.90 (0.29) | 0.89 (0.29) | -0.78 (0.17) | 0.64 (0.16) | 0.60 (0.40) | 0.59 (0.40) | -1.28 (0.30) | 0.45 (0.28) | **2.33 (<0.01)** | **2.35 (<0.01)** | **0.26 (<0.01)** | **1.27 (<0.01)** | 0.59 (0.41) | 0.58 (0.41) | -1.16 (0.27) | 0.49 (0.26) |
| **FAERS-Confusional state** | 1.01 (0.29) | 1.01 (0.29) | **-0.10 (0.01)** | **0.95 (0.02)** | 1.01 (0.29) | 1.01 (0.29) | **-0.11 (0.02)** | **0.94 (0.02)** | 0.82 (0.63) | 0.82 (0.63) | -0.36 (0.09) | 0.79 (0.10) | **1.32 (<0.01)** | **1.32 (<0.01)** | **0.19 (<0.01)** | **1.16 (<0.01)** | 0.81 (0.63) | 0.80 (0.63) | -0.33 (0.08) | 0.81 (0.09) |
| **EMA-Delirium** | **2.09 (<0.01)** | **2.10 (<0.01)** | **-0.08 (0.01)** | **1.08 (0.01)** | 0.63 (0.34) | 0.63 (0.34) | -2.24 (0.41) | 0.30 (0.35) | **1.34 (0.04)** | **1.34 (0.04)** | -0.75 (0.16) | 0.71 (0.12) | 0.79 (0.36) | 0.79 (0.36) | -0.91 (0.20) | 0.62 (0.16) | 0.61 (0.40) | 0.61 (0.40) | -1.74 (0.36) | 0.38 (0.31) |
| **FAERS-Delirium** | 1.00 (0.35) | 1.00 (0.35) | -0.24 (0.05) | 0.88 (0.05) | 1.07 (0.18) | 1.07 (0.18) | **-0.18 (0.03)** | **0.91 (0.03)** | 1.01 (0.32) | 1.01 (0.32) | **-0.24 (0.05)** | **0.88 (0.05)** | 0.99 (0.40) | 0.99 (0.40) | **-0.21 (0.04)** | **0.90 (0.04)** | 0.97 (0.46) | 0.97 (0.46) | -0.24 (0.05) | 0.87 (0.05) |
| **EMA-Delusion** | 0.22 (0.40) | 0.22 (0.40) | 4.06 (0.48) | -0.14 (0.41) | NA | NA | NA | NA | 1.22 (0.14) | 1.22 (0.14) | -1.40 (0.32) | 0.51 (0.24) | **2.01 (<0.01)** | **2.01 (<0.01)** | -0.49 (0.08) | **0.88 (0.04)** | 0.97 (0.22) | 0.97 (0.22) | -1.49 (0.33) | 0.47 (0.26) |

| | | | | | | | | | | | | | | | | | | | | |
|---|---|---|---|---|---|---|---|---|---|---|---|---|---|---|---|---|---|---|---|---|
| **FAERS-Delusion** | 0.96 (0.43) | 0.96 (0.43) | -0.38 (0.10) | 0.81 (0.09) | **1.20 (0.01)** | **1.20 (0.01)** | **-0.13 (0.02)** | **0.96 (0.02)** | 0.82 (0.57) | 0.82 (0.57) | -0.61 (0.17) | 0.69 (0.16) | 0.96 (0.45) | 0.96 (0.45) | -0.30 (0.07) | 0.85 (0.07) | 1.08 (0.15) | 1.08 (0.15) | **-0.19 (0.04)** | **0.92 (0.03)** |
| **EMA-Disorientation** | 0.87 (0.27) | 0.87 (0.27) | -1.21 (0.28) | 0.52 (0.23) | 0.87 (0.28) | 0.87 (0.28) | -1.54 (0.33) | 0.44 (0.28) | 1.20 (0.12) | 1.20 (0.12) | -0.81 (0.18) | 0.67 (0.14) | 0.91 (0.27) | 0.91 (0.27) | -0.70 (0.15) | 0.71 (0.12) | 1.19 (0.11) | 1.19 (0.11) | -0.66 (0.13) | 0.73 (0.10) |
| **FAERS-Disorientation** | 1.08 (0.07) | 1.08 (0.07) | **-0.11 (0.02)** | **0.96 (0.02)** | **1.22 (<0.01)** | **1.23 (<0.01)** | **0.04 (<0.01)** | **1.06 (0.01)** | 0.62 (0.63) | 0.62 (0.63) | -0.85 (0.23) | 0.58 (0.23) | **1.23 (<0.01)** | **1.23 (<0.01)** | **0.05 (<0.01)** | **1.07 (0.01)** | 0.87 (0.60) | 0.87 (0.60) | -0.33 (0.08) | 0.82 (0.08) |
| **EMA-Dissociation** | 0.46 (0.39) | 0.46 (0.39) | -2.86 (0.44) | 0.23 (0.39) | 0.88 (0.25) | 0.88 (0.25) | -2.23 (0.41) | 0.34 (0.34) | NA | NA | NA | NA | **2.35 (<0.01)** | **2.35 (<0.01)** | -0.40 (0.06) | **0.94 (0.03)** | 0.97 (0.22) | 0.97 (0.22) | -1.49 (0.33) | 0.47 (0.26) |
| **FAERS-Dissociation** | 0.68 (0.61) | 0.68 (0.61) | -0.92 (0.25) | 0.56 (0.24) | 0.58 (0.62) | 0.58 (0.62) | -1.24 (0.30) | 0.46 (0.30) | 0.58 (0.62) | 0.58 (0.62) | -1.16 (0.29) | 0.48 (0.28) | **2.63 (<0.01)** | **2.64 (<0.01)** | **0.58 (<0.01)** | **1.57 (<0.01)** | 0.64 (0.62) | 0.64 (0.62) | -0.87 (0.24) | 0.58 (0.23) |
| **EMA-Dizziness** | 0.49 (0.42) | 0.49 (0.42) | -1.34 (0.31) | 0.42 (0.29) | 1.05 (0.15) | 1.05 (0.15) | -0.36 (0.06) | 0.83 (0.06) | 0.40 (0.42) | 0.39 (0.42) | -1.71 (0.35) | 0.33 (0.34) | **2.24 (<0.01)** | **2.28 (<0.01)** | **0.32 (<0.01)** | **1.30 (<0.01)** | 0.61 (0.42) | 0.61 (0.42) | -0.94 (0.21) | 0.55 (0.21) |
| **FAERS-Dizziness** | 0.72 (0.63) | 0.72 (0.63) | -0.50 (0.14) | 0.72 (0.14) | 0.88 (0.62) | 0.88 (0.62) | -0.27 (0.06) | 0.84 (0.07) | 0.50 (0.63) | 0.50 (0.63) | -1.01 (0.26) | 0.51 (0.27) | **2.03 (<0.01)** | **2.05 (<0.01)** | **0.57 (<0.01)** | **1.51 (<0.01)** | 0.80 (0.63) | 0.80 (0.63) | -0.32 (0.08) | 0.81 (0.09) |
| **EMA-Euphoric mood** | 0.46 (0.40) | 0.46 (0.40) | -2.57 (0.42) | 0.25 (0.38) | **2.30 (<0.01)** | **2.30 (<0.01)** | -0.35 (0.06) | **0.92 (0.03)** | 1.22 (0.12) | 1.22 (0.12) | -1.10 (0.26) | 0.58 (0.19) | 1.01 (0.21) | 1.01 (0.21) | -0.80 (0.18) | 0.69 (0.13) | 0.73 (0.33) | 0.74 (0.33) | -1.63 (0.34) | 0.42 (0.30) |
| **FAERS-Euphoric mood** | 0.56 (0.62) | 0.56 (0.62) | -1.18 (0.29) | 0.47 (0.29) | 0.86 (0.53) | 0.86 (0.53) | -0.64 (0.18) | 0.68 (0.17) | **1.27 (<0.01)** | **1.27 (<0.01)** | **-0.08 (0.01)** | **1.00 (0.01)** | 1.08 (0.15) | 1.08 (0.15) | **-0.21 (0.04)** | **0.91 (0.03)** | **1.17 (0.01)** | **1.17 (0.01)** | **-0.14 (0.02)** | **0.96 (0.02)** |
| **EMA-Fall** | 1.02 (0.21) | 1.02 (0.21) | -1.11 (0.26) | 0.56 (0.20) | 0.66 (0.33) | 0.66 (0.33) | -2.18 (0.40) | 0.32 (0.35) | **1.43 (0.01)** | **1.43 (0.01)** | -0.69 (0.14) | 0.74 (0.10) | 0.74 (0.39) | 0.74 (0.39) | -0.98 (0.23) | 0.60 (0.18) | **1.38 (0.01)** | **1.38 (0.01)** | -0.59 (0.11) | 0.78 (0.08) |
| **FAERS-Fall** | **1.48 (<0.01)** | **1.48 (<0.01)** | **0.34 (<0.01)** | **1.29 (<0.01)** | **1.55 (<0.01)** | **1.55 (<0.01)** | **0.41 (<0.01)** | **1.35 (<0.01)** | 0.84 (0.62) | 0.84 (0.62) | -0.36 (0.09) | 0.80 (0.10) | 0.61 (0.63) | 0.61 (0.63) | -0.64 (0.18) | 0.65 (0.19) | 0.98 (0.49) | 0.98 (0.49) | **-0.13 (0.02)** | **0.93 (0.03)** |
| **EMA-Feeling abnormal** | 0.51 (0.40) | 0.51 (0.40) | -1.60 (0.34) | 0.37 (0.31) | 1.04 (0.20) | 1.04 (0.20) | -0.70 (0.15) | 0.68 (0.13) | 0.83 (0.34) | 0.83 (0.34) | -0.90 (0.20) | 0.59 (0.19) | 0.94 (0.27) | 0.94 (0.27) | -0.41 (0.07) | 0.82 (0.07) | **1.71 (<0.01)** | **1.72 (<0.01)** | **0.15 (<0.01)** | **1.19 (<0.01)** |
| **FAERS-Feeling abnormal** | 0.59 (0.63) | 0.59 (0.63) | -0.79 (0.22) | 0.59 (0.22) | 0.85 (0.62) | 0.85 (0.62) | -0.35 (0.08) | 0.80 (0.09) | 1.05 (0.06) | 1.05 (0.06) | **-0.06 (0.01)** | **0.98 (0.02)** | 1.01 (0.29) | 1.01 (0.29) | **-0.08 (0.01)** | **0.96 (0.02)** | **1.40 (<0.01)** | **1.40 (<0.01)** | **0.26 (<0.01)** | **1.21 (<0.01)** |

| | | | | | | | | | | | | | | | | | | | | |
|---|---|---|---|---|---|---|---|---|---|---|---|---|---|---|---|---|---|---|---|---|
| **EMA-Feeling drunk** | 1.00 (0.21) | 1.00 (0.21) | -2.89 (0.44) | 0.29 (0.36) | 1.91 (0.10) | 1.92 (0.10) | -2.51 (0.42) | 0.35 (0.33) | NA | NA | NA | NA | 0.45 (0.38) | 0.45 (0.38) | -2.89 (0.44) | 0.27 (0.37) | 3.33 (<0.01) | 3.33 (<0.01) | -1.23 (0.29) | 0.65 (0.15 |
| **FAERS-Feeling drunk** | 0.52 (0.61) | 0.52 (0.61) | -1.53 (0.34) | 0.39 (0.33) | 0.79 (0.53) | 0.79 (0.53) | -1.02 (0.26) | 0.55 (0.25) | 1.07 (0.28) | 1.07 (0.28) | -0.51 (0.14) | 0.76 (0.12) | 0.86 (0.53) | 0.86 (0.53) | -0.63 (0.18) | 0.70 (0.15) | **1.73 (<0.01)** | **1.73 (<0.01)** | **0.11 (<0.01)** | **1.17 (<0.01)** |
| **EMA-Feeling of relaxation** | NA | NA | NA | NA | NA | NA | NA | NA | Inf (0.47) | Inf (0.47) | -1.37 (0.31) | 0.69 (0.12) | NA | NA | NA | NA | NA | NA | NA | NA |
| **FAERS-Feeling of relaxation** | NA | NA | NA | NA | NA | NA | NA | NA | **3.23 (<0.01)** | **3.23 (<0.01)** | -0.29 (0.07) | **0.98 (0.01)** | **2.11 (<0.01)** | **2.11 (<0.01)** | -0.64 (0.18) | 0.81 (0.09) | NA | NA | NA | NA |
| **EMA-Gait disturbance** | 0.35 (0.39) | 0.35 (0.39) | -2.72 (0.43) | 0.21 (0.40) | 0.67 (0.34) | 0.67 (0.34) | -1.98 (0.38) | 0.34 (0.34) | 1.03 (0.21) | 1.03 (0.21) | -1.03 (0.24) | 0.58 (0.19) | **1.43 (<0.01)** | **1.43 (<0.01)** | -0.33 (0.05) | **0.90 (0.03)** | 1.15 (0.13) | 1.15 (0.13) | -0.69 (0.15) | 0.71 (0.12) |
| **FAERS-Gait disturbance** | **1.10 (0.02)** | **1.10 (0.02)** | **-0.08 (0.01)** | **0.98 (0.02)** | 1.07 (0.12) | 1.07 (0.12) | **-0.13 (0.02)** | **0.94 (0.02)** | 0.81 (0.61) | 0.81 (0.61) | -0.48 (0.13) | 0.74 (0.13) | 0.88 (0.60) | 0.88 (0.60) | -0.29 (0.07) | 0.84 (0.07) | **1.16 (<0.01)** | **1.16 (<0.01)** | **0.00 (0.01)** | **1.03 (0.01)** |
| **EMA-Hallucination** | 0.22 (0.41) | 0.22 (0.41) | -3.28 (0.46) | 0.15 (0.41) | 0.88 (0.28) | 0.88 (0.28) | -1.24 (0.29) | 0.50 (0.24) | 0.79 (0.35) | 0.79 (0.35) | -1.22 (0.28) | 0.50 (0.25) | **1.43 (<0.01)** | **1.43 (<0.01)** | **-0.21 (0.03)** | **0.96 (0.02)** | **1.34 (<0.01)** | **1.34 (<0.01)** | -0.32 (0.05) | **0.89 (0.04)** |
| **FAERS-Hallucination** | **1.10 (0.01)** | **1.10 (0.01)** | **-0.07 (0.01)** | **0.98 (0.01)** | 0.98 (0.41) | 0.98 (0.41) | **-0.24 (0.05)** | 0.87 (0.05) | 1.04 (0.20) | 1.04 (0.20) | **-0.14 (0.02)** | **0.93 (0.03)** | 1.02 (0.28) | 1.02 (0.28) | **-0.13 (0.02)** | **0.94 (0.02)** | 0.89 (0.60) | 0.89 (0.60) | -0.29 (0.07) | 0.84 (0.07) |
| **EMA-Hallucination, auditory** | 0.34 (0.40) | 0.34 (0.40) | -3.17 (0.46) | 0.19 (0.40) | 1.01 (0.21) | 1.01 (0.21) | -1.76 (0.36) | 0.41 (0.30) | 0.88 (0.25) | 0.88 (0.25) | -1.70 (0.35) | 0.42 (0.30) | **2.63 (<0.01)** | **2.64 (<0.01)** | **-0.22 (0.03)** | **1.03 (0.01)** | 0.25 (0.40) | 0.25 (0.40) | -3.48 (0.47) | 0.16 (0.41) |
| **FAERS-Hallucination, auditory** | 0.79 (0.60) | 0.79 (0.60) | -0.07 (0.01) | 0.69 (0.16) | 0.83 (0.57) | 0.83 (0.57) | -0.58 (0.16) | 0.70 (0.16) | 0.88 (0.55) | 0.88 (0.55) | -0.47 (0.13) | 0.76 (0.12) | 0.99 (0.39) | 0.99 (0.39) | **-0.24 (0.05)** | **0.88 (0.05)** | **1.38 (<0.01)** | **1.38 (<0.01)** | **0.11 (<0.01)** | **1.12 (<0.01)** |

| | | | | | | | | | | | | | | | | | | | | |
|---|---|---|---|---|---|---|---|---|---|---|---|---|---|---|---|---|---|---|---|---|
| **EMA-Hallucinations, mixed** | **4.29 (<0.01)** | **4.29 (<0.01)** | -0.44 (0.07) | **0.96 (0.02)** | NA | NA | NA | NA | **2.43 (<0.01)** | **2.43 (<0.01)** | -1.20 (0.28) | 0.62 (0.17) | 0.37 (0.40) | 0.37 (0.40) | -2.67 (0.42) | 0.26 (0.37) | 0.40 (0.34) | 0.40 (0.34) | -3.50 (0.47) | 0.20 (0.40) |
| **FAERS-Hallucinations, mixed** | 1.16 (0.15) | 1.16 (0.15) | -0.35 (0.09) | 0.85 (0.07) | 0.69 (0.57) | 0.69 (0.57) | -1.18 (0.29) | 0.49 (0.28) | **1.82 (<0.01)** | **1.82 (<0.01)** | **0.21 (<0.01)** | **1.24 (<0.01)** | 0.51 (0.62) | 0.51 (0.62) | -1.26 (0.31) | 0.45 (0.30) | **1.22 (0.03)** | **1.22 (0.03)** | **-0.23 (0.05)** | **0.92 (0.03)** |
| **EMA-Hallucination, visual** | 0.18 (0.40) | 0.18 (0.40) | -4.27 (0.48) | 0.12 (0.42) | **1.53 (0.04)** | **1.53 (0.04)** | -1.15 (0.27) | 0.58 (0.19) | 0.46 (0.39) | 0.46 (0.39) | -2.87 (0.44) | 0.23 (0.39) | **1.59 (<0.01)** | **1.59 (<0.01)** | -0.54 (0.09) | 0.84 (0.06) | 1.15 (0.15) | 1.15 (0.15) | -1.10 (0.26) | 0.59 (0.19) |
| **FAERS-Hallucination, visual** | 0.92 (0.51) | 0.92 (0.51) | -0.41 (0.11) | 0.79 (0.10) | **1.51 (<0.01)** | **1.51 (<0.01)** | **0.21 (<0.01)** | **1.21 (<0.01)** | 0.80 (0.59) | 0.80 (0.59) | -0.61 (0.17) | 0.69 (0.16) | 0.85 (0.59) | 0.85 (0.59) | -0.42 (0.11) | 0.78 (0.10) | 1.10 (0.07) | 1.10 (0.07) | **-0.14 (0.02)** | **0.95 (0.02)** |
| **EMA-Incoherent** | NA | NA | NA | NA | 2.30 (0.06) | 2.30 (0.06) | -2.45 (0.42) | 0.36 (0.33) | NA | NA | NA | NA | **5.57 (<0.01)** | **5.57 (<0.01)** | -1.18 (0.28) | 0.72 (0.11) | NA | NA | NA | NA |
| **FAERS-Incoherent** | 1.20 (0.11) | 1.20 (0.11) | -0.31 (0.07) | 0.87 (0.05) | **1.44 (<0.01)** | **1.44 (<0.01)** | **-0.12 (0.02)** | **0.99 (0.01)** | 0.30 (0.62) | 0.41 (0.62) | -2.46 (0.43) | 0.21 (0.43) | **1.52 (<0.01)** | **1.52 (<0.01)** | **0.00 (0.01)** | **1.07 (0.01)** | 0.69 (0.60) | 0.69 (0.60) | -0.92 (0.25) | 0.57 (0.23) |
| **EMA-Mood altered** | 0.13 (0.39) | 0.13 (0.39) | -4.63 (0.49) | 0.10 (0.42) | 1.09 (0.17) | 1.09 (0.17) | -1.46 (0.33) | 0.48 (0.26) | 0.69 (0.33) | 0.69 (0.33) | -1.94 (0.38) | 0.36 (0.33) | **2.09 (<0.01)** | **2.09 (<0.01)** | **-0.25 (0.04)** | **0.99 (0.02)** | 0.80 (0.30) | 0.80 (0.30) | -1.46 (0.32) | 0.46 (0.27) |
| **FAERS-Mood altered** | 0.50 (0.63) | 0.50 (0.63) | -1.24 (0.30) | 0.45 (0.30) | 0.64 (0.62) | 0.64 (0.62) | -0.97 (0.25) | 0.54 (0.25) | 1.11 (0.10) | 1.11 (0.10) | **-0.17 (0.03)** | **0.93 (0.03)** | **1.62 (<0.01)** | **1.62 (<0.01)** | **0.25 (<0.01)** | **1.23 (<0.01)** | 0.96 (0.44) | 0.96 (0.44) | -0.29 (0.07) | 0.85 (0.06) |
| **EMA-Psychotic disorder** | **1.42 (0.01)** | **1.42 (0.01)** | -0.71 (0.15) | 0.73 (0.11) | 0.78 (0.27) | 0.78 (0.27) | -2.01 (0.39) | 0.35 (0.33) | 1.28 (0.11) | 1.28 (0.11) | -0.95 (0.22) | 0.63 (0.16) | 0.76 (0.36) | 0.76 (0.36) | -1.03 (0.24) | 0.59 (0.19) | 1.05 (0.20) | 1.05 (0.20) | -1.04 (0.25) | 0.59 (0.19) |
| **FAERS-Psychotic disorder** | **1.20 (<0.01)** | **1.20 (<0.01)** | **-0.02 (0.01)** | **1.02 (0.01)** | 0.88 (0.55) | 0.88 (0.55) | -0.46 (0.13) | 0.76 (0.12) | **1.29 (<0.01)** | **1.29 (<0.01)** | **0.06 (<0.01)** | **1.08 (0.01)** | 0.80 (0.62) | 0.80 (0.62) | -0.44 (0.11) | 0.77 (0.12) | 0.97 (0.44) | 0.97 (0.44) | -0.24 (0.05) | 0.88 (0.05) |
| **EMA-Somnolence** | **1.23 (<0.01)** | **1.24 (<0.01)** | -0.34 (0.05) | **0.87 (0.05)** | **1.81 (<0.01)** | **1.82 (<0.01)** | **0.08 (<0.01)** | **1.15 (0.01)** | **1.26 (<0.01)** | **1.27 (<0.01)** | -0.34 (0.05) | **0.87 (0.05)** | 0.61 (0.41) | 0.60 (0.41) | -0.85 (0.19) | 0.60 (0.18) | 1.06 (0.15) | 1.06 (0.15) | -0.48 (0.08) | 0.78 (0.08) |
| **FAERS-Somnolence** | 0.97 (0.49) | 0.97 (0.49) | **-0.15 (0.03)** | **0.92 (0.03)** | **1.43 (<0.01)** | **1.43 (<0.01)** | **0.32 (<0.01)** | **1.27 (<0.01)** | 0.90 (0.61) | 0.90 (0.61) | -0.26 (0.06) | 0.85 (0.06) | 0.98 (0.44) | 0.98 (0.44) | **-0.11 (0.02)** | **0.94 (0.02)** | 0.88 (0.62) | 0.88 (0.62) | -0.24 (0.05) | 0.86 (0.06) |

| | | | | | | | | | | | | | | | | | | | | |
|---|---|---|---|---|---|---|---|---|---|---|---|---|---|---|---|---|---|---|---|---|
| **EMA-Substance-induced psychotic disorder** | NA | NA | NA | NA | Inf (0.46) | Inf (0.46) | -2.43 (0.42) | 0.44 (0.28) | NA | NA | NA | NA | NA | NA | NA | NA | NA | NA | NA | NA |
| **FAERS-Substance-induced psychotic disorder** | NA | NA | NA | NA | NA | NA | NA | NA | NA | NA | NA | NA | NA | NA | NA | NA | **6.93 (<0.01)** | **6.93 (<0.01)** | **0.14 (<0.01)** | **1.37 (<0.01)** |
| **EMA-Abnormal thinking** | NA | NA | NA | NA | 0.50 (0.37) | 0.50 (0.37) | -2.78 (0.43) | 0.24 (0.39) | **1.92 (<0.01)** | **1.92 (<0.01)** | -0.34 (0.05) | **0.92 (0.03)** | **1.43 (<0.01)** | **1.43 (<0.01)** | -0.47 (0.08) | 0.85 (0.05) | 1.03 (0.21) | 1.03 (0.21) | -1.06 (0.25) | 0.58 (0.20) |
| **FAERS-Abnormal thinking** | 0.48 (0.63) | 0.48 (0.63) | -1.27 (0.31) | 0.44 (0.31) | 0.84 (0.58) | 0.84 (0.58) | -0.53 (0.15) | 0.72 (0.14) | 0.90 (0.55) | 0.90 (0.55) | -0.41 (0.11) | 0.78 (0.10) | **1.23 (<0.01)** | **1.24 (<0.01)** | **0.02 (0.01)** | **1.05 (0.01)** | **1.37 (<0.01)** | **1.37 (<0.01)** | **0.13 (<0.01)** | **1.13 (<0.01)** |

Boldface denotes signals based on FDR<0.05; Minimum number of events to compute signal statistics = 5 for all measures.

Abbreviations: EB05 = 5% quantile of the posterior distribution of the empirical Bayesian geometric mean (estimated FDR); EMA: European Medicines Agency; FAERS: Food and Drug Administration Adverse Event Reporting System; FDR = false discovery rate; IC025= 2.5% quantile of the posterior distribution of information component (estimated FDR); NA = not available = less than 5 events for this pair; PRR= observed relative risks (estimated FDR); ROR= observed odds ratios (estimated FDR).
