# Supplementary material for: A Focus on Abuse/Misuse and Withdrawal Issues with Selective Serotonin Reuptake Inhibitors (SSRIs): Analysis of Both the European EMA and the US FAERS Pharmacovigilance Databases
Source: Pharmaceuticals (Basel). 2022 May 1;15(5):565. doi: 10.3390/ph15050565 (PMC9146999; doi:10.3390/ph15050565)
Supplement: Supplementary file 1 [file pharmaceuticals-15-00565-s001.zip › TableS4_R3.pdf]

Table S4. Estimated US prevalence estimate of prescription antidepressant use in past month according to the National Health and Nutrition Examination Survey (NHANES) data (2003-2018).

Years	2003-2004	2005-2006	2007-2008	2009-2010	2011-2012	2013-2014	2015-2016	2017-2018
**Estimate (95% CI) antidepressant use**	5.6% (4.9-6.5)	5.3% (4.7-6.0)	5.7% (4.8-6.7)	5.2% (4.1-6.5)	6.5% (5.2-8.2)	6.9% (6.0-7.9)	6.2% (5.1-7.5)	6.4% (5.5-7.4)

Estimate = prevalence estimate (number of individuals who have used one of the antidepressants (ADs) citalopram, escitalopram, fluoxetine, paroxetine, and sertraline in the past month. Antidepressant use = reported using citalopram, escitalopram, fluoxetine, paroxetine, and/or sertraline in the past month. CI: confidence interval; US: United States
